# Retirement as risk or relief? The role of timing in mental, physical and cognitive health effects of retirement

**DOI:** 10.1007/s10433-025-00898-2

**Published:** 2025-12-10

**Authors:** Isabelle Hansson, Anne Ingeborg Berg, Pär Bjälkebring, Sandra Buratti, Linda B. Hassing, Valgeir Thorvaldsson, Boo Johansson

**Affiliations:** Department of Psychology, University of Gothenburg, Box 500, 405 30 Gothenburg, Sweden

**Keywords:** Retirement transition, Health, Retirement age, Job satisfaction, Involuntary retirement, Longitudinal study

## Abstract

Retirement is a major life event that can significantly impact health and well-being in later life. In this study, we evaluated how effects of retirement on mental, physical, and cognitive health vary depending on retirement age, pre-retirement job satisfaction, and degree of voluntariness in the transition. Using nine annual measurement waves from the longitudinal population-based HEalth, Aging, and Retirement Transitions in Sweden (HEARTS) study (*N* = 5,913, age 60–74), we applied linear-mixed effects models to assess changes in life satisfaction, quality of life, depressive symptoms, disease burden, reasoning ability, and memory over the retirement transition. Results showed average improvements across health domains post-retirement, with more pronounced benefits among those who retired early. High pre-retirement job satisfaction and involuntary retirement increased the risk of negative changes in mental health, particularly among those who retired later. The findings illustrate how health effects of retirement are driven by push (out of work) and pull (into retirement) factors. While extended working lives can benefit those who find meaning and value in work, delaying retirement for individuals who would benefit from leaving the workforce may increase the public health burden.

**Supplementary Information:**

The online version contains supplementary material available at 10.1007/s10433-025-00898-2.

## Introduction

Retirement from work is a significant life transition that brings substantial changes to daily life. As a marker of entry into a new life stage, it requires adaptation to age-related changes and shifts in routines, activities, and social life. How do people cope with these changes, and how do they influence health and well-being? Which aspects of health are more likely to change, and what determines the direction and magnitude of those changes? How retirement impacts health and well-being has been of interest in both academic and public debate for decades (van Solinge & Hansson 2025). In populations with growing proportions of older adults, negative health consequences represent a major public health concern (World Health Organization 2015). With increasing pressures for extended working lives to ensure the sustainability of welfare systems, higher retirement ages may both alleviate and increase negative health consequences for individuals and societies (OECD 2023). Understanding the conditions under which retirement is associated with positive or negative health changes is essential for developing strategies to address the challenges and opportunities of rapidly aging populations.

The aim of the current study was to determine how health effects of retirement vary depending on age at retirement, pre-retirement job satisfaction, and voluntariness of the transition. We conceptualize health as a multidimensional construct—encompassing mental, physical, and cognitive capacities—to generate a comprehensive understanding of how retirement affects various aspects of health and the extent to which similar or differential patterns arise. Building on previous research on the heterogeneity in retirement pathways (Madero-Cabib et al. 2023; Turek et al. 2024), we expect a selection effect reflected by better health among those who retire later (Fisher et al. 2016). We further expect that health effects of retirement depend not only on the timing of the event, but also on contextual factors shaping the transition (Henning et al. 2016; van der Heide et al. 2013; Zulka et al. 2019). In countries with a flexible pension scheme like Sweden, with pension eligibility from the age of 61 (until 2020) and legal right to remain employed until 69 (since 2023), people may choose to retire early to enjoy life in retirement or feel forced to do so because of poor health or working conditions (Brockmann et al. 2009; Topa et al. 2018). Individuals may also decide to continue working because they enjoy their job or because of limited financial resources (Barnes-Farrel, 2003). Health effects of retirement can therefore be driven by both push (out of work) and pull (into retirement) factors (Shultz et al. 1998).

In a trade-off between work and retirement, we expect that those who retire early to a larger extent do so because they find their current situation unsatisfactory or because they are longing for a life free of work demands (Wang & Shultz 2010). Retirement may in this sense bring a relief from work-related demands while allowing for recovery and investment in activities outside of work (Grøtting & Lillebø, 2020; Topa et al. 2018). For those who retire later, on the other hand, work is expected to have a greater personal meaning, either in terms of intrinsic values or through extrinsic rewards (Baxter et al. 2021). Retirement may therefore to a larger extent be associated with losses. Based on this reasoning, we anticipate that early retirement is associated with poorer pre-retirement health, but more beneficial effects of retirement.

To further disentangle health effects of early and late retirement, both pre-retirement job satisfaction and the degree of voluntariness in the transition are considered. While individuals with low job satisfaction are expected to show more positive effects of retirement (Kubicek et al. 2011), satisfaction with one’s job is an important driver for continuing working (Browne et al. 2019). For these individuals, retirement may be particularly challenging. Building on a trade-off between push and pull mechanisms, we assume that individuals who choose to retire early do not necessarily have to struggle to find meaning and satisfaction in retirement (Barnes-Farrel & Matthews, 2009). It is thus plausible to find satisfaction in one’s job while at the same time experiencing a desire for retirement (Shultz et al. 1998). Those who want to continue working but are being pushed out of work because they have reached a certain age may find it difficult to replace work-related rewards in retirement (Topa & Valero 2017). We therefore anticipate that higher job satisfaction is associated with more negative health effects of retirement, particularly among those who retire later.

While lack of control in the timing of retirement can have negative health effects in both early and late retirement (Xue et al. 2022), the effect may be less pronounced if it enables recovery from work-related demands (Shultz et al. 1998). Negative effects of involuntary early retirement due to poor health could thereby be mitigated by a health recovery after retirement (Brockmann et al. 2009; Grøtting & Lillebø, 2020; Jokela et al. 2010). Forced late retirement can, on the other hand, to a larger extent reflect lack of opportunity to continue working due to the enforcement of pension regulations (Hyde & Dingemans 2017). For these individuals, work-related benefits can be difficult to find and replace elsewhere (Baxter et al. 2021). Based on this reasoning, we anticipate that involuntarily retirement is associated with negative health effects of retirement, particularly among those who retire later.

In summary, we propose the following three hypotheses:

H1. Health effects of retirement vary by age; individuals who retire early show poorer pre-retirement health and more positive effects of retirement compared to those retiring later.

H2. Job satisfaction moderates the association between retirement age and health; individuals with higher job satisfaction show more negative health effects of retirement, and this association is more pronounced among those who retire later.

H3. The voluntariness of the transition moderates the association between retirement age and health; individuals who retire involuntarily show more negative health effects of retirement, and this association is more pronounced among those who retire later.

### Study context

The hypothesized health effects of retirement are examined in a population-based sample of Swedish adults transitioning to retirement. With nine annual measurement waves, covering a total age range of 60–74, the Health, Aging, and Retirement Transitions in Sweden (HEARTS) study (Lindwall et al. 2017) constitutes one of few longitudinal cohort studies on retirement that allow detailed evaluation of heterogeneity in effects of retirement on key indicators of mental (life satisfaction, quality of life, depressive symptoms), physical (disease burden), and cognitive (reasoning ability, memory) health. The data was collected between the years 2015–2023, a period during which the retirement age in Sweden has gradually increased (Masic & Granseth 2025). The participants were unaffected by changes in the age of pension eligibility (relevant if born 1959 or later), but a substantial proportion (those born 1952 or later) had the opportunity to delay their retirement due to changes in the Employment Protection Act (Masic & Granseth 2025). The possible age range of retirement in the HEARTS sample thereby spans from 61 to 69, with options to combine pension with work (enabling gradual and multiple transitions) and to extend work beyond the age of 69 (if permitted by employer). While the average retirement age for this cohort is 65, both early, late, and gradual retirement is prevalent, reflecting increasing diversity of pathways to retirement (Masic & Granseth 2025). The Swedish public pension follows a defined-contribution design (supplemented by a guarantee pension for low lifetime earners), with age-related incentives that reward late (higher monthly income) and penalize early (lower monthly income) exits (König & Sjögren Lindquist 2016). By evaluating how health effects of retirement vary depending on retirement age, pre-retirement job satisfaction, and voluntariness of the transition, the present study seeks to elucidate conditions under which retirement is associated with increases or decreases in mental, physical, and cognitive health.

## Method

### Participants

We utilize data from nine annual measurement waves of the HEARTS study (Lindwall et al. 2017), a Swedish longitudinal population-based survey of people born between 1949 and 1955 (age 60–66 at baseline). The first survey was distributed in 2015 (*N* = 5,913; response rate 39.4%), with annual follow-ups conducted in 2016 (*N* = 4,651, 78.7% of baseline sample), 2017 (*N* = 4,320, 73.1%), 2018 (*N* = 4,033, 68.2%), 2019 (*N* = 3,935, 66.6%), 2020 (*N* = 3,914, 66.2%), 2021 (*N* = 3,660, 61.9%), 2022 (*N* = 3,305, 55.9%), and 2023 (*N* = 3,160, 53.4%). Details on the study design, measurement domains, and previous publications can be found at https://osf.io/wcbxu/.

The total number of person-time observations in the sample is 36,891, the average number of waves completed per participant is 6.24 (*SD* = 2.88), and more than half of the participants (55.9%;* n* = 3,307) have completed seven or more waves (for more details, see Table 1). In this study, we included all participants who provided valid data on variables of interest in the analyses (for further details, see analysis section). Exact number of individuals and observations in each analysis are presented in the result tables.

**Table 1:** Descriptive statistics of demographic characteristics, retirement status, and health variables (life satisfaction, quality of life, depressive symptoms, disease burden, reasoning ability, and memory) across waves

	Year								
	2015	2016	2017	2018	2019	2020	2021	2022	2023
*N*^a^	5,913	4,651	4,320	4,033	3,935	3,914	3,660	3,305	3,160
Age (range 60–74), *M* (*SD*)	63.09 (2.01)	64.15 (2.01)	65.14 (2.00)	66.11 (2.00)	67.08 (2.00)	68.07 (2.01)	69.04 (2.00)	70.05 (2.00)	71.02 (2.00)
Gender (% women)	53.9	54.6	54.0	53.8	54.3	53.9	54.2	54.0	53.8
Education (% tertiary)	40.8	43.0	43.9	43.4	43.9	44.5	46.0	46.0	46.0
Relationship status (% with partner)	80.9	81.15	81.18	80.4	79.9	79.5	78.8	77.9	77.5
Employment status at baseline (% unemployed)	7.9								
Retirement status (range 1–4), *M* (*SD*)	1.83 (1.25)	2.25 (1.35)	2.59 (1.35)	2.88 (1.29)	3.14 (1.17)	3.38 (0.99)	3.55 (0.84)	3.62 (0.76)	3.67 (0.70)
Not retired (%)	65.9	48.9	36.0	25.7	16.9	8.8	4.3	2.5	1.5
Partially retired, do not identify as retiree (%)	7.7	10.6	11.8	12.4	11.3	11.0	9.9	9.4	8.5
Partially retired, identify as retiree (%)	4.5	7.4	9.4	10.4	12.6	13.6	12.0	11.5	11.8
Fully retired (%)	21.9	33.1	42.8	51.5	59.2	66.6	73.8	76.6	78.2
Life satisfaction (range 5–35), *M* (*SD*)	24.18 (6.98)	24.37 (6.81)	24.49 (6.93)	24.76 (7.11)	24.92 (6.94)	25.18 (6.83)	25.83 (6.75)	25.88 (6.79)	25.88 (6.71)
Quality of life (range 12–48), *M* (*SD*)	38.18 (6.21)	38.38 (6.08)	38.47 (6.11)	39.00 (6.22)	39.05 (6.21)	38.97 (6.11)	39.18 (6.10)	39.25 (6.05)	39.23 (6.10)
Depressive symptoms (range 0–33), *M* (*SD*)	4.43 (4.49)	4.19 (4.35)	4.06 (4.31)	3.91 (4.25)	3.98 (4.16)	4.38 (4.40)	4.19 (4.23)	4.15 (4.34)	4.04 (4.24)
Disease burden (range 0–46), *M* (*SD*)	5.88 (3.73)	5.87 (3.70)	5.85 (3.63)	6.01 (3.64)	6.28 (3.74)	6.27 (3.78)	6.43 (3.79)	6.55 (3.71)	6.68 (3.82)
Reasoning ability (range 0–12), *M* (*SD*)	–	5.39 (1.69)	5.57 (1.69)	5.72 (1.69)	5.81 (1.70)	6.06 (1.60)	6.07 (1.66)	6.13 (1.66)	6.10 (1.66)
Memory (range 0–20), *M* (*SD*)	16.39 (2.38)	16.84 (2.41)	–	17.26 (2.29)	17.59 (2.16)	17.66 (2.19)	﻿–	﻿–	17.69 (2.30)
Job satisfaction^b^ (range 1–7), *M* (*SD*)	5.85 (1.17)	5.80 (1.22)	5.86 (1.18)	5.89 (1.18)	5.99 (1.12)	6.19 (1.02)	6.28 (0.92)	6.34 (0.34)	6.38 (0.88)
Involuntary transition^b^ (range 0–2),* M* (*SD*)	0.36 (0.66)	0.40 (0.69)	0.41 (0.69)	0.41 (0.70)	0.38 (0.70)	0.40 (0.69)	0.28 (0.59)	0.28 (0.59)	0.34 (0.65)

*Note*. ^a^37.6% (*n* = 2,223) completed all nine waves, 10.4% (*n* = 615) completed 8 waves, 7.9% (*n* = 469) completed 7 waves, 6.9% (*n* = 411) completed 6 waves, 5.9% (*n* = 347) completed 5 waves, 7.4% (*n* = 438) completed 4 waves, 7.2% (*n* = 424) completed 3 waves, 7.9% (*n* = 470) completed 2 waves, and 8.7% (*n* = 516) only completed the baseline assessment. ^b^Treated as time-invariant (person mean) in the analyses

### Measures

#### Retirement status

Retirement status was assessed in each wave with the question “Are you retired (i.e., have started to receive old-age pension)?”, with the following response alternatives: (1) No, (2) Yes, but still working and do not consider myself a retiree, (3) Yes, still working but consider myself a retiree, and (4) Yes, “full-time retiree”. Retirement is treated as a continuous variable, ranging from not retired (1) to fully retired (4). Response alternative 2 and 3 are considered as partial retirement (working while drawing pension), where 2 refers to the initial step into retirement (pension withdrawal) and 3 as identification with life as a retiree (subjective retirement). To separate within-person effects of retirement (i.e., change in retirement status) from between-person differences in retirement status (i.e., workers vs. retirees), two variables are calculated. First, a time-constant (between-person) variable was generated by calculating the individual mean across measurement points. This variable was centered on the sample mean. Second, a time-varying (within-person) variable was generated by subtracting the person mean from the individual response at each measurement point (i.e., person mean centering).

#### Health indicators

Health was assessed with six different indicators. Mental health was measured through indices of life satisfaction, quality of life, and depressive symptoms. Physical health was measured with an index of disease burden, and cognitive health was measured by tests of reasoning ability and memory.

*Life satisfaction* was measured in each wave with the Satisfaction With Life Scale (Diener et al. 1985). The scale consists of five items (e.g., “I am satisfied with my life”) measured on a 7-point scale, ranging from strongly disagree (1) to strongly agree (7). Cronbach’s alpha ranged from 0.92 to 0.93 across measurement waves. Scores were summarized to an index (range 5–35) and standardized (z-score) on the baseline distribution.

*Quality of life* was measured in each wave with the Quality of Life Scale (CASP-12; von dem Knesebeck et al. 2005). The scale consists of 12 items (e.g., “I feel that my life has meaning”) measured on a 4-point scale, ranging from strongly disagree (1) to strongly agree (4). Cronbach’s alpha ranged from 0.85 to 0.86 across measurement waves. Scores were summarized to an index (range 12–48) and standardized (z-score) on the baseline distribution.

*Depressive symptoms* were measured in each wave with an 11-item version of the Center for Epidemiologic Studies Depression (CES-D) Scale (Radloff 1977). The participants were asked to rate how often during the last week they had experienced each symptom (e.g., “I felt depressed”). Response alternatives ranged from never/rarely (less than 1 day) (0) to aways/almost always (5–7 days) (3). Cronbach’s alpha ranged from 0.81 to 0.82 across measurement waves. Scores were summarized to an index (range 0–33) and standardized (z-score) on the baseline distribution.

*Disease burden* was measured in each wave with an index of 23 common diseases (e.g., hypertension, cardiovascular diseases, diabetes, asthma, stroke) and health complaints (e.g., back pain, pain in neck or shoulders, headache, visual impairment, reduced ability to walk). Mental health symptoms (e.g., tiredness, anxiety, memory problems) were also assessed but excluded from the index as not to confound the assessment of physical health. Response alternatives were no (0), yes, symptoms and mild (or no) discomfort (1), and yes, symptoms and severe discomfort (2). Scores were summarized to an index (range 0–46) and standardized (z-score) on the baseline distribution.

*Reasoning ability* was measured with a short version of Raven’s Advanced Progressive matrices (Arthur & Day 1994). With a total time limit of three minutes, 12 different matrices were displayed. The matrices consisted of visual geometric designs with a missing piece. Participants were given six to eight choices to choose from and were asked to fill in the missing piece. Number of correct answers were summarized (range 0–12) and standardized (z-score) on the baseline distribution. The test was administered in the online version of the survey and included in wave two to nine.

*Memory* was measured with Thurstone’s Picture Memory task (Thurstone & Thurstone 1949), a recognition test consisting of 20 pictures that were subsequently presented to the participants for five seconds each (encoding phase). Directly after this phase, participants were asked to identify each of the 20 previously viewed pictures when they were shown together with three other related pictures (i.e., of the same or a similar object in a different position) that had not previously been shown (recognition phase). Number of correct answers were summarized (range 0–20) and standardized (z-score) on the baseline distribution. The test was administered in the online version of the survey and included in wave 1, 2, 4, 5, 6, and 9.

#### Job satisfaction and involuntary transition

*Job satisfaction* was assessed in each wave (for participants indicating that they were not yet retired) with the item “How satisfied are you with your current job overall?”. Response alternatives ranged from very dissatisfied (1) to very satisfied (7). To accommodate missing data post-retirement, job satisfaction was treated as a between-person variable by calculating the person mean across measurement waves (ICC = 0.56).

*Involuntary transition* to retirement was assessed in each wave (for participants indicating that they were retired) with the item “Did you choose the timing of your retirement, or did you feel forced (due to health, employer, etc.)?”. In all waves, the response alternatives ranged from “I chose completely by myself” to “I could not choose at all”, but the number of options varied from five (wave 1–6) to three (wave 7–9). The middle options (score 2–4) in wave one to six were therefore combined to form a harmonized category of partial involuntariness. The resulting measure was coded as completely voluntary (0), partially involuntary (1), and completely involuntary (2). To accommodate missing data pre-retirement, involuntary transition was treated as a between-person variable by calculating the person mean across measurement waves (ICC = 0.64).

#### Covariates

Age (in years) was included both as time-varying (across measurement waves) and time-constant (age at baseline) to facilitate estimation of within-person changes over time (slope) while accounting for between-person (cohort) differences at baseline. Gender (male = 0, female = 1), education (primary/secondary = 0, tertiary = 1), baseline employment status (employed = 0, unemployed = 1), and relationship status (single = 0, in a relationship = 1) in each wave were included as covariates.

### Statistical analyses

Effects of retirement on the six health indicators (life satisfaction, quality of life, depressive symptoms, disease burden, reasoning ability, and memory) were evaluated in separate linear-mixed effects models using the lme4 package (Bates et al. 2015) in R (version 4.3.2; R Core Team, 2023).

#### Within- and between-person effects of retirement

Retirement status was included as both time-varying (level 1) and as time-constant (level 2) predictors of health. Effects of the time-varying (within-person) variable can thereby be interpreted as the average within-person change in health when individuals transition from work to retirement. The time-constant (between-person) variable of retirement status, on the other hand, indicates the average difference in health between workers and retirees. Age (as both time-varying, centered on 65, and time-constant, centered on the sample mean at baseline), gender, education, baseline employment status, and relationship status (time-varying) were included as covariates. The two age variables differentiate trajectories over time (with increasing age) from cohort differences (in baseline age). To evaluate individual variability in effects, the level 1 covariates of retirement status and age were included as random slope effects.

#### Health effects by retirement age

The hypothesis that individuals who retire early show poorer pre-retirement health and more positive effects of retirement compared to those retiring later (H1) was evaluated by including interaction terms between age (level 1) and retirement (level 1 and 2). Within-person effects of retirement by age can thereby be differentiated from average (between-person) differences between workers and retirees at a certain age. To evaluate transition-specific effects, we preformed a sensitivity analysis with varying definitions of retirement. In these models, the retirement status variable was recoded to differentiate transitions from 1 to 2 (pension withdrawal), 2–3 (subjective retirement), and 3–4 (stop working).

#### Moderating effects of job satisfaction and involuntary transition

The hypothesis that individuals with higher job satisfaction show more negative health effects of retirement, and that this association is more pronounced among those who retire later (H2) was evaluated by including a three-way interaction between retirement, age, and job satisfaction. Finally, the hypothesis that individuals who retire involuntarily show more negative health effects of retirement, and that this association is more pronounced among those who retire later (H3) was evaluated by including a three-way interaction between retirement, age, and involuntary transition.

#### Sample selection and inference criteria

In models evaluating H1, all individuals with valid scores on retirement status were included (*N* = 5,875). For H2, only participants with pre-retirement assessment of job satisfaction were included (*N* = 4,041). For H3, only participants with (post-retirement) reports on involuntary transition were included (*N* = 4,656). Depending on data availability in the dependent variable, the number of participants included in each model varied across health indicators (exact *N* indicated in tables). In all models, the alpha level was restricted to 0.008 using the Bonferroni correction (0.05/6) to account for multiple testing.

## Results

Table 1 shows descriptives statistics on demographic characteristics, retirement status, and health variables at each measurement wave. Most participants were not yet retired (65.9%) at the first measurement wave in 2015 and fully retired (78.2%) at wave nine in 2023. A transition matrix of the number of transitions between each retirement status category shows that the majority transition from not retired to fully retired, but that a substantial proportion gradually withdraw from work (Supplementary Material, Table S1). Pre-retirement job satisfaction was relatively high (*M* = 5.87, *SD* = 1.05) and only a small proportion (6.3%) reported completely involuntary retirement (partially involuntary = 28.1%, completely voluntary = 65.6%). Variances, covariances, and correlations of study variables are presented in the supplementary material (Table S2). Intraclass correlation coefficients showed a relatively high within-person stability in life satisfaction (ICC = 0.72), quality of life (ICC = 0.74), and disease burden (ICC = 0.81) over time and more variability in depressive symptoms (ICC = 0.66), reasoning ability (ICC = 0.51), and memory (ICC = 0.50).

### Within- and between-person effects of retirement

Results of the linear-mixed effects models showed an average within-person increase in life satisfaction (*b* = 0.04, *p* < 0.001), quality of life (*b* = 0.05, *p* < 0.001), and memory (*b* = 0.02, *p* = 0.001), and an average decrease in depressive symptoms (*b* = -0.04, *p* < 0.001) and disease burden (*b* = -0.02, *p* < 0.001) after retirement (see Table 2, Retirement WP). No significant within-person change in relation to retirement was found for reasoning ability (*b* = 0.01, *p* = 0.44). Random effects showed relatively large variability in the within-person effect of retirement on mental (*SD* = 0.13) and physical (*SD* = 0.11) health but smaller differences for cognition (*SD* = 0.01–0.05). Between-person effects showed that retirees on average reported higher life satisfaction (*b* = 0.06, *p* < 0.001) than workers, but no significant differences in quality of life (*b* = 0.01, *p* = 0.36), depressive symptoms (*b* = -0.01, *p* = 0.40), disease burden (*b* = 0.04, *p* = 0.02), reasoning ability (*b* = -0.01, *p* = 0.52), and memory (*b* = 0.02, *p* = 0.17; see Table 2, Retirement BP).

**Table 2:** Conditional effects of retirement on life satisfaction, quality of life, depressive symptoms, disease burden, reasoning ability, and memory

	Life satisfaction			Quality of life			Depressive symptoms			Disease burden			Reasoning ability			Memory		
	Est	95%CI	*p*	Est	95%CI	*p*	Est	95%CI	*p*	Est	95%CI	*p*	Est	95%CI	*p*	Est	95%CI	*p*
*Fixed effects*																		
Intercept	−0.23	−0.28, −0.18	< .001	−0.11	−0.16, −0.06	< .001	0.18	0.13, 0.22	< .001	0.12	0.07, 0.17	< .001	−0.01	−0.07, 0.05	.744	−0.01	−0.07, 0.05	.660
Age at baseline	−0.02	−0.03, −0.004	.013	−0.005	−0.02, 0.01	.499	−0.02	−0.03, −0.002	.024	−0.04	−0.06, −0.03	< .001	−0.13	−0.14, −0.11	< .001	−0.08	−0.10, −0.07	< .001
Age (time−varying)	0.02	0.01, 0.02	< .001	0.005	0.001, 0.01	.009	0.01	0.01, 0.01	< .001	0.04	0.04, 0.05	< .001	0.06	0.05, 0.06	< .001	0.07	0.06, 0.07	< .001
Retirement WP	0.04	0.03, 0.05	< .001	0.05	0.04, 0.06	< .001	−0.04	−0.05, −0.03	< .001	−0.02	−0.03, −0.01	< .001	0.01	−0.01, 0.02	.439	0.02	0.01, 0.04	.001
Retirement BP	0.06	0.03, 0.09	< .001	0.01	−0.02, 0.04	.362	−0.01	−0.04, 0.02	.396	0.04	0.01, 0.07	.019	−0.01	−0.04, 0.02	.521	0.02	−0.01, 0.05	.172
Gender^a^	0.03	−0.01, 0.08	.130	0.05	0.001, 0.09	.044	0.07	0.03, 0.11	.002	0.02	−0.03, 0.07	.346	−0.22	−0.27, −0.18	< .001	0.19	0.14, 0.24	< .001
Education^b^	0.08	0.03, 0.12	.001	0.08	0.03, 0.12	.001	−0.03	−0.07, 0.01	.192	−0.27	−0.32, −0.22	< .001	0.29	0.25, 0.34	< .001	0.24	0.20, 0.29	< .001
Relationship status^c^	0.35	0.31, 0.38	< .001	0.21	0.18, 0.24	< .001	−0.37	−0.41, −0.34	< .001	−0.05	−0.08, −0.01	.008	0.05	0.002, 0.10	.043	−0.005	−0.05, 0.04	.845
Employment status at baseline^d^	−0.61	−0.70, −0.53	< .001	−0.69	−0.78, −0.61	< .001	0.63	0.55, 0.71	< .001	0.81	0.71, 0.91	< .001	−0.14	−0.24, −0.05	.004	−0.19	−0.28, −0.09	< .001

*Random effects*	*SD*	Δχ^2^(3)^e^	*p*	*SD*	Δχ^2^(3)^e^	*p*	*SD*	Δχ^2^(3)^e^	*p*	*SD*	Δχ^2^(3)^e^	*p*	*SD*	Δχ^2^(3)^e^	*p*	*SD*	Δχ^2^(3)^e^	*p*
Intercept	0.80			0.82			0.74			0.87			0.68			0.69		
Age	0.07	603.25	< .001	0.07	496.59	< .001	0.07	458.84	< .001	0.07	620.17	< .001	0.05	24.62	< .001	0.06	84.09	< .001
Retirement WP	0.13	142.61	< .001	0.13	156.69	< .001	0.13	112.29	< .001	0.11	102.82	< .001	0.05	4.64	.199	0.01	2.18	.536
Residual	0.48			0.47			0.54			0.39			0.69			0.66		
AIC	64,439.85			61,713.80			68,156.92			43,437.24			52,903.51			43,395.43		
BIC	64,574.43			61,847.90			68,291.19			43,567.18			53,031.26			43,519.81		
*N* / observations	5,502 / 33,218			5,479 / 32,255			5,550 / 32,588			5,173 / 24,871			4,369 / 21,686			4,631 / 17,563		

Dependent variables standardized (z-score) on baseline distribution to facilitate interpretation. WP = within-person effect, BP = between-person effect, AIC = Akaike Information Criterion, BIC = Bayesian Information Criterion, CI = confidence interval. ^a^Male = 0, Female = 1. ^b^Primary/secondary = 0, Tertiary = 1. ^c^No partner = 0, With partner = 1. ^d^Employed = 0, Unemployed = 1. ^e^Likelihood-ratio test of random effect (df = 3)

### Health effects by retirement age

Interaction effects of retirement and age showed that the within-person effect of retirement was more pronounced among those who retired earlier (as indicated by a reduction of the effect with increasing age; see Table 3). Significant interactions were found on life satisfaction (*b* = -0.01, *p* = 0.001), quality of life (*b* = -0.01, *p* < 0.001), depressive symptoms (*b* = 0.01, *p* < 0.001), disease burden (*b* = 0.01, *p* = 0.001), reasoning ability (*b* = -0.01, *p* < 0.001), and memory (*b* = -0.02, *p* < 0.001). Evaluation of the simple effect slopes showed significant within-person improvements in life satisfaction (*b* = 0.06, *p* < 0.001), quality of life (*b* = 0.10, *p* < 0.001), depressive symptoms (*b* = -0.10, *p* < 0.001), disease burden (*b* = -0.04, *p* < 0.001), reasoning ability (*b* = 0.06, *p* < 0.001), and memory (*b* = 0.10, *p* < 0.001) for individuals retiring at age 61, and no or negative effects at age 69 (see Supplementary Material, Table S3). Specifically, for those retiring at age 69, there was no significant change in life satisfaction (*b* = 0.02, *p* = 0.03), quality of life (*b* = -0.01, *p* = 0.28), depressive symptoms (*b* = 0.02, *p* = 0.01), or disease burden (*b* = 0.002, *p* = 0.79), but declines in reasoning ability (*b* = -0.04, *p* = 0.002) and memory (*b* = -0.06, *p* < 0.001).

**Table 3:** Interaction effects of retirement and age on life satisfaction, quality of life, depressive symptoms, disease burden, reasoning ability, and memory

	Life satisfaction			Quality of life			Depressive symptoms			Disease burden			Reasoning ability			Memory		
	Est	95%CI	*p*	Est	95%CI	*p*	Est	95%CI	*p*	Est	95%CI	*p*	Est	95%CI	*p*	Est	95%CI	*p*
*Fixed effects*																		
Intercept	−0.21	−0.26, −0.16	< .001	−0.08	−0.13, −0.03	.001	0.15	0.10, 0.19	< .001	0.11	0.06, 0.16	< .001	−0.003	−0.06, 0.06	.932	0.01	−0.05, 0.07	.748
Age at baseline	−0.02	-0.03, -0.01	.005	−0.01	−0.03, 0.003	.115	−0.01	−0.02, 0.01	.248	-0.04	−0.06, −0.02	< .001	−0.13	−0.15, −0.12	< .001	−0.09	−0.11, -0.08	< .001
Age (time-varying)	0.02	0.02, 0.03	< .001	0.01	0.01, 0.02	< .001	0.003	−0.001, 0.01	.126	0.04	0.04, 0.04	< .001	0.06	0.06, 0.07	< .001	0.07	0.07, 0.08	< .001
Retirement WP	0.04	0.03, 0.05	< .001	0.04	0.03, 0.05	< .001	-0.04	−0.05, −0.03	< .001	−0.02	−0.03, -0.01	< .001	0.01	−0.002, 0.03	.104	0.02	0.005, 0.03	.008
Retirement BP	0.07	0.04, 0.09	< .001	0.02	−0.01, 0.05	.275	−0.02	−0.05, 0.01	.197	0.04	0.004, 0.07	.027	−0.01	−0.05, 0.02	.459	0.02	−0.01, 0.06	.135
Gender^a^	0.04	−0.01, 0.08	.103	0.05	0.01, 0.10	.027	0.06	0.02, 0.11	.003	0.02	−0.03, 0.07	.384	−0.22	−0.27, −0.17	< .001	0.19	0.15, 0.24	< .001
Education^b^	0.07	0.03, 0.12	.002	0.07	0.02, 0.11	.005	−0.02	−0.06, 0.02	.321	−0.26	−0.31, −0.21	< .001	0.30	0.25, 0.34	< .001	0.24	0.20, 0.29	< .001
Relationship status^c^	0.35	0.31, 0.38	< .001	0.21	0.18, 0.24	< .001	−0.37	−0.41, -0.34	< .001	−0.05	−0.08, −0.01	.009	0.05	0.001, 0.10	.045	−0.004	−0.05, 0.05	.880
Employment status at baseline^d^	−0.61	−0.69, -0.52	< .001	−0.68	−0.77, −0.59	< .001	0.62	0.54, 0.70	< .001	0.81	0.71, 0.91	< .001	−0.14	−0.24, −0.04	.006	−0.18	−0.28, −0.08	< .001
Retirement WP × Age	−0.01	−0.01, −0.002	.001	−0.01	−0.02, −0.01	< .001	0.01	0.01, 0.02	< .001	0.01	0.002, 0.01	.001	−0.01	−0.02, −0.01	< .001	−0.02	−0.03, −0.02	< .001
Retirement BP × Age	−0.01	−0.02, −0.01	< .001	−0.02	−0.02, −0.02	< .001	0.02	0.01, 0.02	< .001	0.01	0.002, 0.01	.001	0.001	−0.01, 0.01	.949	−0.004	−0.01, 0.001	.140
*Random effects (SD)*																		
Intercept	0.80			0.82			0.74			0.87			0.68			0.69		
Age	0.07			0.06			0.07			0.07			0.05			0.07		
Retirement WP	0.13			0.13			0.13			0.11			0.05			0.01		
Residual	0.48			0.47			0.54			0.39			0.69			0.66		
AIC	64,373.33			61,518.73			68,024.36			43,419.06			52,887.85			43,333.36		
BIC	64,524.73			61,669.59			68,175.41			43,565.24			53,031.56			43,473.29		
*N* / observations	5,502 / 33,218			5,479 / 32,255			5,550 / 32,588			5,173 / 24,871			4,369 / 21,686			4,631 / 17,563		

Dependent variables standardized (z-score) on baseline distribution to facilitate interpretation. WP = within-person effect, BP = between-person effect, AIC = Akaike Information Criterion, BIC = Bayesian Information Criterion, CI = confidence interval. ^a^Male = 0, Female = 1. ^b^Primary/secondary = 0, Tertiary = 1. ^c^No partner = 0, With partner = 1. ^d^Employed = 0, Unemployed = 1

Results additionally showed a reduction in average (between-person) differences between workers and retirees with increasing age for life satisfaction (*b* = -0.01, *p* < 0.001), quality of life (*b* = -0.02, *p* < 0.001), and depressive symptoms (*b* = 0.02, *p* < 0.001), but increasing differences in disease burden (*b* = 0.01, *p* = 0.001; see Table 3). No significant interaction was found for reasoning ability (*b* = 0.001, *p* = 0.95) and memory (*b* = -0.004, *p* = 0.14). While average mental health differences between workers and retirees decreased with increasing age, individuals still working at age 69 showed higher quality of life (*b* = -0.06, *p* < 0.001), fewer depressive symptoms (*b* = 0.05, *p* < 0.001), and lower disease burden (*b* = 0.06, *p* = 0.001) compared to those already retired (see Supplementary Material, Table S3).

**Fig. 1:**
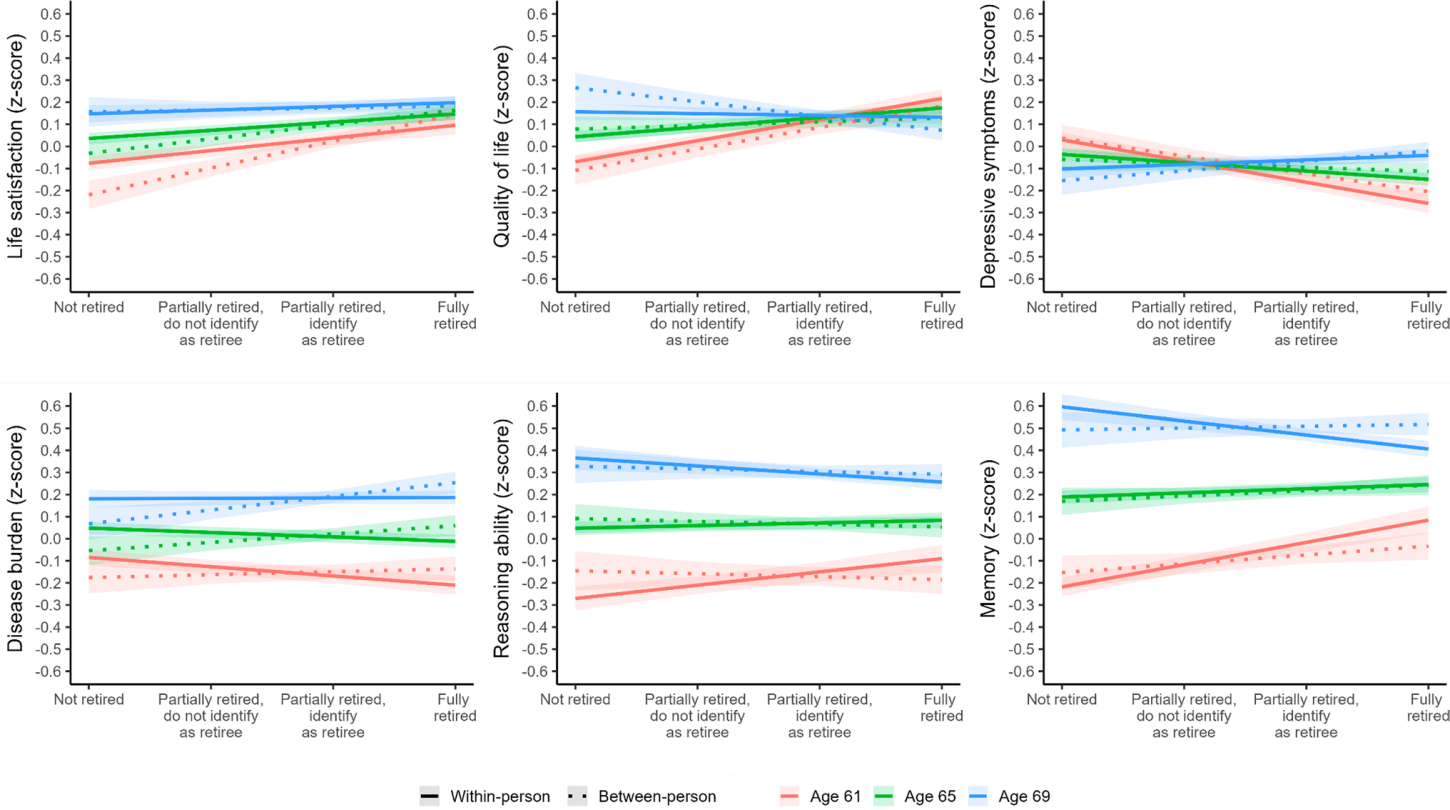
Estimated within- and between-person fixed effects of retirement at age 61, 65, and 69 on life satisfaction, quality of life, depressive symptoms, disease burden, reasoning ability, and memory

Figure 1 illustrates the predicted within- and between-person effects of retirement at age 61, 65, and 69. In support of H1, results show that individuals who retire early demonstrate poorer mental (life satisfaction, quality of life, depressive symptoms) and cognitive (reasoning ability, memory) health prior to retirement, and more positive effects of retirement, compared to individuals retiring later. For disease burden, the positive effect of retirement (in terms of reduced disease burden) was also more pronounced among those retiring early, but they showed lower disease burden prior to retirement compared to individuals retiring later.

Sensitivity analysis of differences in health effects depending on the type of transition showed that models with retirement as a continuous predictor generally generated better model fit (ΔAIC and ΔBIC > 20) than models where retirement was identified by pension withdrawal, subjective retirement, or complete withdrawal from work (see Supplementary Material, Tables S4−﻿S9). For cognition, the difference in model fit was relatively small (ΔAIC and ΔBIC ≤ 7). Comparing the three models with varying definitions of retirement, subjective retirement appeared to produce the best fit for all health variables except for reasoning ability (where pension withdrawal and subjective retirement showed comparable fit), suggesting that the subjective component of feeling retired is of relevance when evaluating health effects of retirement. The overall pattern of associations however remained the same.

### Health effects by retirement age and job satisfaction

**Fig. 2:**
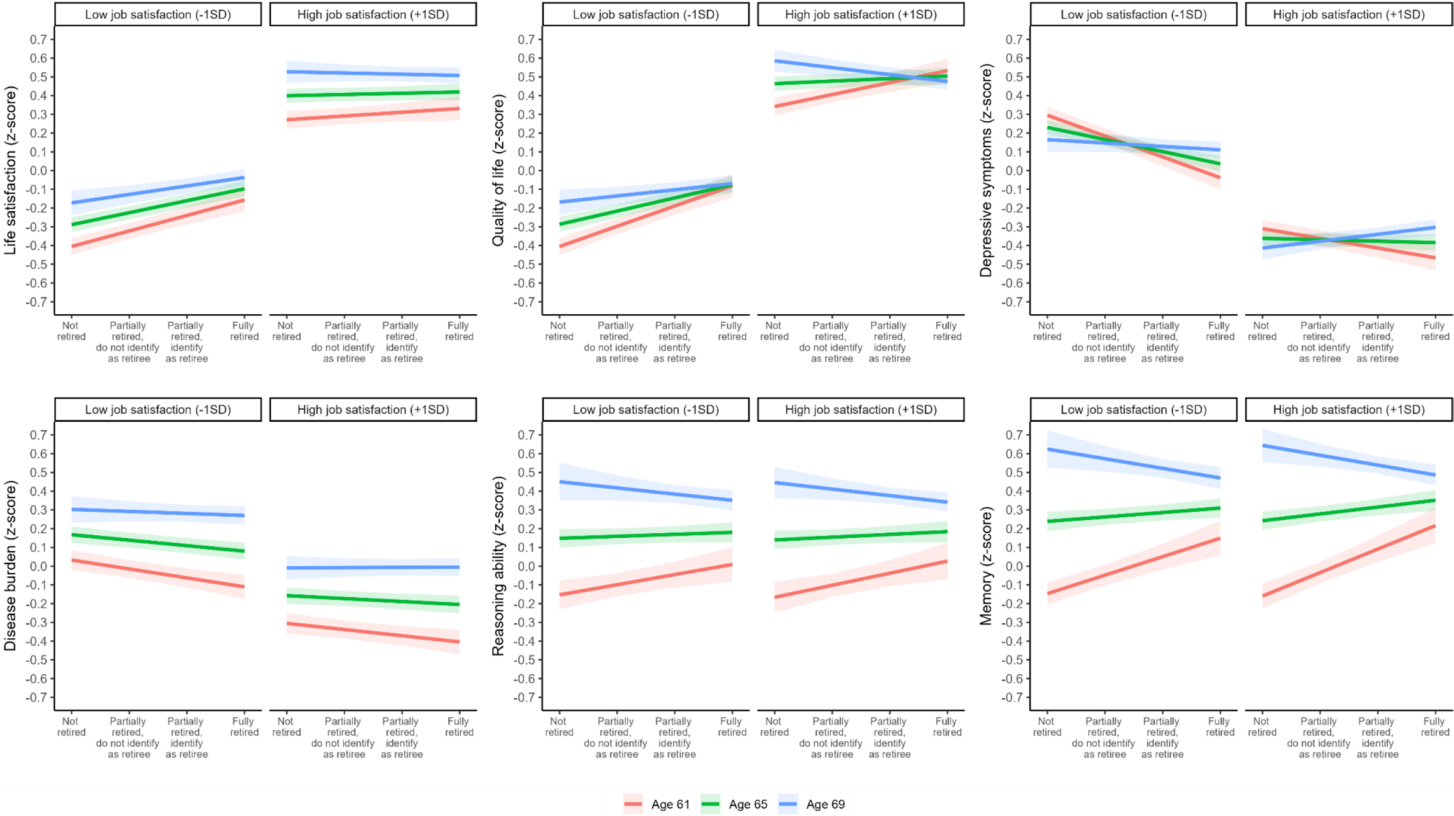
Estimated within-person fixed effects of retirement (at age 61, 65, and 69) by job satisfaction (-1SD vs. + 1SD) on life satisfaction, quality of life, depressive symptoms, disease burden, reasoning ability, and memory

Moderating effects of job satisfaction in the association between retirement age and health showed no significant three-way interactions (see Supplementary Material, Table S10). Individuals with higher job satisfaction showed higher life satisfaction (*b* = 0.30, *p* < 0.001) and quality of life (*b* = 0.33, *p* < 0.001), less depressive symptoms (*b* = -﻿0.25, *p* < 0.001), and lower disease burden (*b* = -0.15, *p* < 0.001), but no significant differences in reasoning ability (*b* = -﻿0.001, *p* = 0.93) and memory (*b* = 0.01, *p* = 0.44). With partial support of H2, higher pre-retirement job satisfaction was related to less improvements in life satisfaction (*b* = -﻿0.03, *p* < 0.001), quality of life (*b* = -﻿0.03, *p* < 0.001), and depressive symptoms (*b* = 0.03, *p* < 0.001) after retirement. Figure 2 shows the predicted within-person effects of retirement (at age 61, 65, and 69) for individuals with high (+ 1SD) and low (-1SD) job satisfaction. Evaluation of simple effect slopes showed that individuals with low job satisfaction showed improvements in life satisfaction and quality of life regardless of age at retirement (61, 65, or 69; see Supplementary Material, Table S3). For depressive symptoms and disease burden, the improvement among those with low job satisfaction was present only at age 61 and 65. For individuals retiring at age 69, high job satisfaction was related to an increase in depressive symptoms (*b* = 0.04, *p* ﻿= 0.002) and a decline in quality of life (*b* = -﻿0.04, *p* = 0.001).

### Health effects by retirement age and involuntary transition

**Fig. 3:**
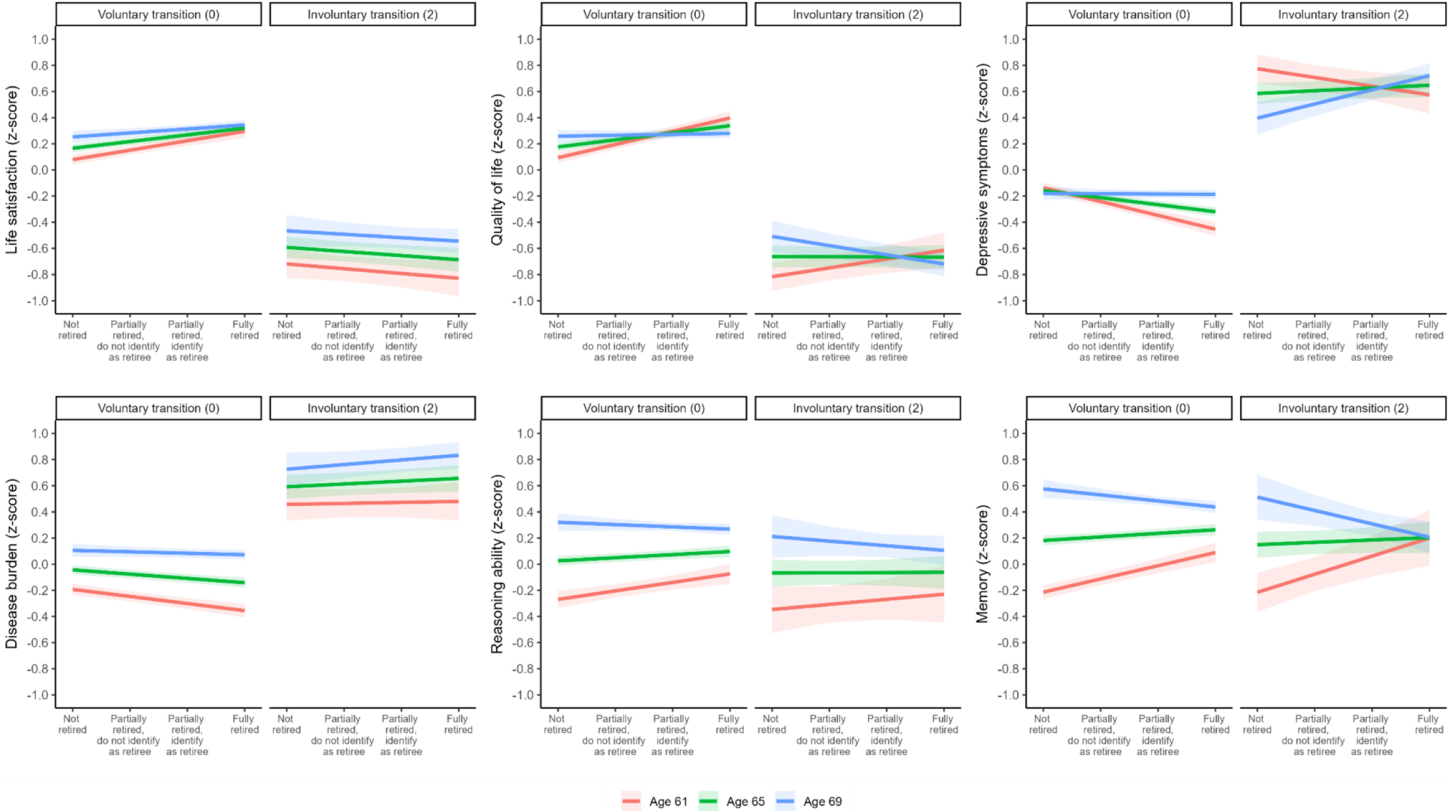
Estimated within-person fixed effects of retirement (at age 61, 65, and 69) by voluntariness of the transition (voluntary vs. involuntary) on life satisfaction, quality of life, depressive symptoms, disease burden, reasoning ability, and memory

Moderating effects of involuntary transition in the association between retirement age and health revealed no significant three-way interactions (see Supplementary Material, Table S11). Individuals with involuntary transitions showed lower life satisfaction (*b* = ﻿-0.44, *p* < 0.001) and quality of life (*b* = -0.46, *p* < 0.001), more depressive symptoms (*b* = 0.43, *p* < 0.001), higher disease burden (*b* = 0.36, *p* < 0.001), but no significant differences in reasoning ability (*b* = ﻿-0.06, *p* = 0.02) and memory (*b* = ﻿-0.02, *p* = 0.38). With partial support of H3, involuntary retirement was related to less improvements in life satisfaction (*b* = -0.04, *p* < 0.001), quality of life (*b* = -0.03, *p* < 0.001), depressive symptoms (*b* = 0.04, *p* < 0.001), and disease burden (*b* = 0.03, *p* = 0.001). Figure 3 shows the predicted within-person effects of retirement (at age 61, 65, and 69) for individuals with voluntary and involuntary transitions. Evaluation of simple effect slopes showed that individuals with voluntary transitions demonstrated improvements in life satisfaction regardless of age at retirement (61, 65 or 69; see Supplementary Material, Table S3). For quality of life, depressive symptoms, and disease burden, improvement among those with voluntary transitions were observed only at age 61 and 65. For individuals retiring at age 69, involuntary retirement was associated with an increase in depressive symptoms (*b* = 0.11, *p* < 0.001) and a decline in quality of life (*b* = ﻿-0.07, *p* = 0.002).

## Discussion

The aim of this study was to evaluate how mental, physical, and cognitive health effects of retirement vary depending on retirement age, pre-retirement job satisfaction, and degree of voluntariness in the transition. Results from nine annual measurement waves in a population-based sample of Swedish adults transitioning to retirement showed average improvements in life satisfaction, quality of life, depressive symptoms, disease burden, and memory after retirement. Results further showed that positive health effects are more pronounced among individuals who retire early, which largely can be attributed to poorer pre-retirement health. High pre-retirement job satisfaction and involuntary retirement increased the risk of negative mental health effects, particularly among those who retire later. The findings illustrate how health effects of retirement are driven by push (out of work) and pull (into retirement) factors, and that higher retirement ages can both increase and alleviate the public health burden of aging populations.

By evaluating effects of retirement on central indicators of mental, physical, and cognitive health, the present study facilitates a comprehensive understanding of how retirement affects various aspects of health. The results show similar patterns of improvements after retirement across measures, although mental health effects were more pronounced. The finding that individuals retiring early showed poorer health prior to retirement and stronger health improvements after retirement (H1) adds to previous reports of heterogeneity in health effects of retirement (Brockmann et al. 2009; Grøtting & Lillebø, 2020; Nielsen 2019; Qvist 2023). Our results suggest that health inequalities in retirement can be attributed to pre-retirement levels rather than within-person changes across the transition. The finding of better health and no (or negative) health changes among those retiring later suggest that retirement primarily benefits those who are unable or unwilling to extend their work life. While the contrasting finding of higher disease burden among those retiring later likely reflects an accumulation of age-related biological contingencies, the between-person comparison of workers and retirees at age 69 confirms the anticipated selection effect of better health among those who continue to work into older ages (Brockmann et al. 2009; Fisher et al. 2016). A forced delay of retirement for individuals who would benefit from leaving the workforce may therefore increase health inequalities in older age (Grøtting & Lillebø, 2020; König et al. 2018; Nielsen 2019; Qvist 2023).

Results further support the anticipated differences in health effects depending on pre-retirement job satisfaction (H2) and degree of voluntariness in the transition (H3). While high job satisfaction and involuntary retirement are well-established risk factors in the process of adjusting to life in retirement (van Solinge & Hansson 2025), the present study suggests that they primarily play a role for mental health effects of retirement, and particularly among those who retire later. While involuntary early retirement was related to less health improvements compared to voluntary retirement, results suggest that early involuntary retirement reflect a necessity to leave one’s job that does not necessarily generate poorer health. Instead, it can bring a sense of relief from work-related demands that counteract continued health deterioration (Grøtting & Lillebø, 2020; van den Bogaard et al. 2016). The finding that early retirement from satisfying jobs was related to stability (or improvements) rather than declines in health reflect a desire for retirement that is stronger than the desire for work-related rewards (Okamoto et al. 2023). Adjustment to life in retirement may therefore be less difficult for these individuals (Henning et al. 2019). For those who retire later, on the other hand, work-related losses may be more pronounced (Topa & Valero 2017), as reflected by increases in depressive symptoms and declines in quality of life. Forced retirement for individuals who struggle to replace the loss of work-related rewards therefore constitutes a risk factor for developing mental health problems after retirement. While enabling employment beyond the age of 69 may alleviate some of these challenges (Hansson et al. 2018b, 2023), efforts to support adjustment to the loss of the work role remain crucial for a significant proportion of retirees (Hansson et al. 2019; Henning et al. 2021).

For disease burden, the findings add to previous reports of negative consequences of retirement due to poor health (Hershey & Henkens 2014; König et al. 2019) but underscores the presence of general health inequalities (Brockmann et al. 2009). While involuntary retirement was related to higher overall disease burden, it did not increase health burden after retirement. For reasoning ability and memory, the general finding of improvements in early retirement and declines in late retirement suggests that the timing of retirement is of primary importance for understanding how retirement affects cognitive health. Overall, the results indicate that physical and cognitive health, as operationalized in this study, are less sensitive to changes in response to retirement compared to indicators of mental health.

## Limitations and future directions

The findings are of relevance for understanding when, how, and why retirement affects various aspects of health. Several limitations should, however, be acknowledged. First, while the longitudinal design allows for separation of within-person changes relative to between-person differences over time, health-related selection into early versus late retirement may not be fully captured by our estimation of differences in pre-retirement health. Second, disease burden was based on self-reported symptoms and illnesses, and cognitive tests were conducted online, which may reduce reliability. Third, job satisfaction and involuntary transition were treated as time-constant variables to facilitate estimation of health changes across the transition. As both may vary over time (Henning et al. 2024; Zacher & Rudolph 2017), future studies should consider evaluating how changes in job satisfaction and voluntariness of the transition (particularly for individuals who undergo multiple transitions) relate to health effects of retirement. Finally, although the recruitment of participants in HEARTS was population-based, selective attrition (e.g., higher retention rate among individuals with higher education; Hansson et al. 2018a) may limit the generalizability of our findings. Sweden's relatively generous pension system and flexible retirement options may also limit the ability to generalize findings to countries with more restrictive retirement policies.

While the present cohort was unaffected by the recent increases in the age of pension eligibility in Sweden, results suggest that positive health effects are most pronounced among those who retire early. The extent to which similar or differential patterns are found in later born cohorts who are expected to work longer remains to be investigated. The findings nevertheless underscore the need to invest resources to promote healthy pathways to retirement (Henkens 2022). For individuals who are willing and able to work into old age, the loss of employment may elevate health risks after retirement. Striking a balance between extending work life and providing options for early exit is therefore a critical task for employers and policy makers.

## Supplementary Information

Below is the link to the electronic supplementary material.

Supplementary file1 (DOCX 102 KB)

## Data Availability

Data from the HEARTS study can be made available upon request and in accordance with applicable laws. Data requests should be addressed to hearts@psy.gu.se.
