# Supplementary material for: Retirement as risk or relief? The role of timing in mental, physical and cognitive health effects of retirement
Source: Eur J Ageing. 2025 Dec 10;23(1):1. doi: 10.1007/s10433-025-00898-2 (PMC12779816; doi:10.1007/s10433-025-00898-2)
Supplement: Supplementary file 1 — Supplementary file1 (DOCX 102 KB) [file 10433_2025_898_MOESM1_ESM.docx]

**Supplementary material**

**Retirement as Risk or Relief?**

**The Role of Timing in Mental, Physical and Cognitive Health Effects of Retirement**

**Table S1**

*Frequency of transitions between retirement status categories across measurement waves*

|  | To | | | |
| --- | --- | --- | --- | --- |
| From | Not retired | Partially retired, do not identify as retiree | Partially retired, identify as retiree | Fully retired |
| Not retired | 5,327 | 859 | 453 | 1,493 |
| Partially retired, do not identify as retiree | 237 | 1,592 | 540 | 467 |
| Partially retired, identify as retiree | 15 | 346 | 1,632 | 864 |
| Fully retired | 68 | 132 | 481 | 13,585 |

*Note*. *N* = 5,875. Diagonal: no change in retirement status across two consecutive waves. Number of individuals constantly not retired: *n* = 644; Constantly partially retired, do not identify as retiree: *n* = 351; Constantly partially retired, identify as retiree: *n* = 404; Constantly fully retired: *n* = 1,113; At least one transition: *n* = 3,363.

**Table S2**

*Correlations (above diagonal), variances (diagonal), and covariances (below diagonal) of demographic characteristics (age, gender, education, relationship status), retirement status, health variables (life satisfaction, quality of life, depressive symptoms, disease burden, reasoning ability, and memory), job satisfaction, and involuntary transition*

|  |  | 1. | 2. | 3. | 4. | 5. | 6. | 7. | 8. | 9. | 10. | 11. | 12. | 13. | 14. |
| --- | --- | --- | --- | --- | --- | --- | --- | --- | --- | --- | --- | --- | --- | --- | --- |
| 1. | Age^a^ | 10.71 | 0.01 | 0.002 | -0.02 | 0.63 | -0.05 | 0.10 | 0.06 | -0.03 | 0.07 | 0.02 | 0.14 | 0.09 | 0.02 |
| 2. | Gender^b^ | 0.02 | 0.25 | 0.09 | -0.11 | 0.04 | 0.05 | -0.004 | <0.001 | 0.06 | 0.004 | -0.09 | 0.10 | -0.04 | 0.02 |
| 3. | Education^c^ | 0.004 | 0.02 | 0.24 | 0.02 | -0.07 | -0.09 | 0.05 | 0.05 | -0.01 | -0.14 | 0.14 | 0.13 | 0.05 | -0.04 |
| 4. | Relationship status^ad^ | -0.03 | -0.02 | 0.004 | 0.16 | 0.01 | -0.09 | 0.23 | 0.16 | -0.20 | -0.07 | 0.03 | -0.01 | 0.09 | -0.14 |
| 5. | Retirement status^a^ | 2.69 | 0.02 | -0.04 | 0.01 | 1.70 | -0.02 | 0.09 | 0.05 | -0.03 | 0.08 | -0.02 | 0.08 | -0.05 | -0.02 |
| 6. | Baseline employment status | -0.05 | 0.01 | -0.01 | -0.01 | -0.01 | 0.07 | -0.16 | -0.17 | 0.16 | 0.20 | -0.04 | -0.05 | -0.09 | 0.29 |
| 7. | Life satisfaction^a^ | 2.18 | -0.01 | 0.16 | 0.63 | 0.85 | -0.27 | 47.76 | 0.75 | -0.63 | -0.31 | 0.07 | 0.08 | 0.32 | -0.28 |
| 8. | Quality of life^a^ | 1.10 | <0.001 | 0.14 | 0.38 | 0.38 | -0.26 | 31.62 | 37.81 | -0.68 | -0.40 | 0.07 | 0.07 | 0.35 | -0.30 |
| 9. | Depressive symptoms^a^ | -0.37 | 0.14 | -0.03 | -0.35 | -0.20 | 0.17 | -18.81 | -17.93 | 18.69 | 0.37 | -0.06 | -0.06 | -0.29 | 0.28 |
| 10. | Disease burden^a^ | 0.89 | 0.004 | -0.25 | -0.11 | 0.37 | 0.17 | -7.96 | -8.91 | 5.84 | 13.94 | -0.04 | -0.03 | -0.16 | 0.24 |
| 11. | Reasoning ability^a^ | 0.11 | -0.08 | 0.11 | 0.02 | -0.04 | -0.02 | 0.81 | 0.70 | -0.42 | -0.22 | 2.85 | 0.23 | -0.001 | -0.04 |
| 12. | Memory^a^ | 1.02 | 0.12 | 0.16 | -0.01 | 0.25 | -0.03 | 1.26 | 1.03 | -0.54 | -0.25 | 0.88 | 5.51 | 0.01 | -0.05 |
| 13. | Job satisfaction | 0.29 | -0.02 | 0.02 | 0.04 | -0.07 | -0.01 | 2.15 | 2.09 | -1.19 | -0.58 | -0.002 | 0.03 | 1.10 | -0.12 |
| 14. | Involuntary transition | 0.04 | 0.01 | -0.01 | -0.03 | -0.01 | 0.05 | -1.10 | -1.03 | 0.68 | 0.49 | -0.04 | -0.06 | -0.07 | 0.36 |

*Note*. Below diagonal: covariances. Diagonal: variances. Above diagonal: correlation coefficients. ^a^Time-varying. ^b^Male = 0, Female = 1. ^c^Primary/secondary = 0, Tertiary = 1. ^d^No partner = 0, With partner = 1.

**Table S3**

*Estimated simple effect slopes of retirement on life satisfaction, quality of life, depressive symptoms, disease burden, reasoning ability, and memory by age, job satisfaction, and voluntariness of the transition*

|  |  | Life satisfaction | | | Quality of life | | | Depressive symptoms | | | Disease burden | | | Reasoning ability | | | Memory | | |
| --- | --- | --- | --- | --- | --- | --- | --- | --- | --- | --- | --- | --- | --- | --- | --- | --- | --- | --- | --- |
|  |  | Est. | *SE* | *p* | Est. | *SE* | *p* | Est. | *SE* | *p* | Est. | *SE* | *p* | Est. | *SE* | *p* | Est. | *SE* | *p* |
|  | | | | | | | | | | | | | | | | | | | |
| *Within- and between-person effects of retirement by age (as depicted in Figure 1)* | | | | | | | | | | | | | | | | | | | |
| Within-person effect | Age 61 | 0.06 | 0.01 | <.001 | 0.10 | 0.01 | <.001 | -0.10 | 0.01 | <.001 | -0.04 | 0.01 | <.001 | 0.06 | 0.01 | <.001 | 0.10 | 0.01 | <.001 |
|  | Age 65 | 0.04 | 0.01 | <.001 | 0.04 | 0.01 | <.001 | -0.04 | 0.01 | <.001 | -0.02 | 0.01 | <.001 | 0.01 | 0.01 | .104 | 0.02 | 0.01 | .008 |
|  | Age 69 | 0.02 | 0.01 | .027 | -0.01 | 0.01 | .281 | 0.02 | 0.01 | .014 | 0.002 | 0.01 | .790 | -0.04 | 0.01 | .002 | -0.06 | 0.01 | <.001 |
| Between-person effect | Age 61 | 0.12 | 0.02 | <.001 | 0.10 | 0.02 | <.001 | -0.08 | 0.02 | <.001 | 0.01 | 0.02 | .479 | -0.01 | 0.02 | .547 | 0.04 | 0.02 | .044 |
|  | Age 65 | 0.07 | 0.02 | <.001 | 0.02 | 0.02 | .276 | -0.02 | 0.01 | .197 | 0.04 | 0.02 | .027 | -0.01 | 0.02 | .459 | 0.02 | 0.02 | .135 |
|  | Age 69 | 0.01 | 0.02 | .589 | -0.06 | 0.02 | <.001 | 0.05 | 0.02 | .005 | 0.06 | 0.02 | .001 | -0.01 | 0.02 | .518 | 0.01 | 0.02 | .663 |
|  | | | | | | | | | | | | | | | | | | | |
| *Within-person effect of retirement by job satisfaction and age (as depicted in Figure 2)* | | | | | | | | | | | | | | | | | | | |
| Low job satisfaction  (-1SD) | Age 61 | 0.08 | 0.01 | <.001 | 0.11 | 0.01 | <.001 | -0.11 | 0.01 | <.001 | -0.05 | 0.01 | <.001 | 0.05 | 0.02 | .005 | 0.10 | 0.02 | <.001 |
|  | Age 65 | 0.06 | 0.01 | <.001 | 0.07 | 0.01 | <.001 | -0.07 | 0.01 | <.001 | -0.03 | 0.01 | <.001 | 0.01 | 0.01 | .306 | 0.02 | 0.01 | .029 |
|  | Age 69 | 0.05 | 0.01 | <.001 | 0.03 | 0.01 | .007 | -0.02 | 0.01 | .175 | -0.01 | 0.01 | .328 | -0.03 | 0.02 | .103 | -0.05 | 0.02 | .010 |
| High job satisfaction (+1SD) | Age 61 | 0.02 | 0.01 | .536 | 0.06 | 0.01 | <.001 | -0.05 | 0.01 | <.001 | -0.03 | 0.01 | .008 | 0.06 | 0.02 | .004 | 0.13 | 0.02 | <.001 |
|  | Age 65 | 0.01 | 0.01 | .338 | 0.01 | 0.01 | .051 | -0.01 | 0.01 | .310 | -0.02 | 0.01 | .023 | 0.02 | 0.01 | .194 | 0.04 | 0.01 | .002 |
|  | Age 69 | -0.01 | 0.01 | .092 | -0.04 | 0.01 | .001 | 0.04 | 0.01 | .002 | 0.001 | 0.01 | .897 | -0.04 | 0.02 | .041 | -0.05 | 0.02 | .004 |
|  | | | | | | | | | | | | | | | | | | | |
| *Within-person effect of retirement by voluntariness of the transition and age (as depicted in Figure 3)* | | | | | | | | | | | | | | | | | | | |
| Voluntary transition (0) | Age 61 | 0.07 | 0.01 | <.001 | 0.10 | 0.01 | <.001 | -0.11 | 0.01 | <.001 | -0.05 | 0.01 | <.001 | 0.07 | 0.02 | <.001 | 0.10 | 0.01 | <.001 |
|  | Age 65 | 0.05 | 0.01 | <.001 | 0.05 | 0.01 | <.001 | -0.05 | 0.01 | <.001 | -0.03 | 0.01 | <.001 | 0.02 | 0.01 | .007 | 0.03 | 0.01 | .001 |
|  | Age 69 | 0.03 | 0.01 | .001 | 0.01 | 0.01 | .448 | -0.002 | 0.01 | .835 | -0.01 | 0.01 | .228 | -0.02 | 0.02 | .229 | -0.05 | 0.02 | .003 |
| Involuntary transition (2) | Age 61 | -0.04 | 0.03 | .148 | 0.06 | 0.03 | .008 | -0.07 | 0.03 | .016 | 0.01 | 0.03 | .780 | 0.04 | 0.04 | .415 | 0.14 | 0.04 | .001 |
|  | Age 65 | -0.03 | 0.01 | .022 | -0.001 | 0.01 | .921 | 0.02 | 0.02 | .157 | 0.02 | 0.01 | .128 | 0.002 | 0.02 | .938 | 0.02 | 0.02 | .403 |
|  | Age 69 | -0.03 | 0.02 | .244 | -0.07 | 0.02 | .002 | 0.11 | 0.03 | <.001 | 0.04 | 0.02 | .121 | -0.04 | 0.05 | .310 | -0.10 | 0.04 | .006 |

**Table S4**

*Sensitivity analysis with varying definitions of retirement (pension withdrawal, subjective retirement, stop working) in the effects on life satisfaction*

|  | Life satisfaction | | | | | | | | |
| --- | --- | --- | --- | --- | --- | --- | --- | --- | --- |
|  | Pension withdrawal^a^ | | | Subjective retirement^b^ | | | Stop working^c^ | | |
|  | Est. | 95% CI | *p* | Est. | 95% CI | *p* | Est. | 95% CI | *p* |
| *Fixed effects* | | | | | | | | | |
| Intercept | -0.22 | -0.27, -0.17 | <.001 | -0.21 | -0.26, -0.16 | <.001 | -0.21 | -0.26, -0.16 | <.001 |
| Age at baseline | -0.03 | -0.04, -0.01 | .001 | -0.02 | -0.03, -0.01 | .004 | -0.02 | -0.03, -0.002 | .021 |
| Age (time-varying) | 0.02 | 0.02, 0.03 | <.001 | 0.02 | 0.02, 0.03 | <.001 | 0.02 | 0.02, 0.03 | <.001 |
| Retirement WP | 0.10 | 0.08, 0.13 | <.001 | 0.10 | 0.08, 0.12 | <.001 | 0.10 | 0.07, 0.12 | <.001 |
| Retirement BP | 0.25 | 0.15, 0.34 | <.001 | 0.17 | 0.10, 0.25 | <.001 | 0.12 | 0.05, 0.19 | .001 |
| Gender^d^ | 0.04 | -0.003, 0.09 | .066 | 0.04 | -0.01, 0.08 | .108 | 0.04 | -0.01, 0.08 | .095 |
| Education^e^ | 0.07 | 0.02, 0.11 | .003 | 0.07 | 0.03, 0.12 | .002 | 0.07 | 0.02, 0.11 | .003 |
| Relationship status^f^ | 0.35 | 0.31, 0.38 | <.001 | 0.35 | 0.31, 0.38 | <.001 | 0.35 | 0.31, 0.38 | <.001 |
| Employment status at baseline^g^ | -0.59 | -0.68, -0.51 | <.001 | -0.61 | -0.70, -0.52 | <.001 | -0.62 | -0.70, -0.53 | <.001 |
| Retirement WP × Age | <0.001 | -0.01, 0.01 | .979 | -0.02 | -0.03, -0.01 | <.001 | -0.02 | -0.03, -0.01 | <.001 |
| Retirement BP × Age | -0.03 | -0.05, -0.02 | <.001 | -0.04 | -0.05, -0.03 | <.001 | -0.04 | -0.04, -0.03 | <.001 |
| *Random effects (SD)* | | | | | | | | | |
| Intercept | 0.80 | | | 0.80 | | | 0.80 | | |
| Age | 0.07 | | | 0.07 | | | 0.07 | | |
| Retirement WP | 0.33 | | | 0.32 | | | 0.31 | | |
| Residual | 0.48 | | | 0.48 | | | 0.48 | | |
| AIC | 64,426.39 | | | 64,339.81 | | | 64,514.09 | | |
| BIC | 64,577.78 | | | 64,551.20 | | | 64,665.48 | | |

*Note*. Dependent variable standardized (z-score) on baseline distribution to facilitate interpretation. WP = within-person effect, BP = between-person effect, AIC = Akaike Information Criterion, BIC = Bayesian Information Criterion, CI = confidence interval. ^a^Retirement defined by transition from 1 to 2/3/4 in the retirement status variable. ^b^Retirement defined by transition from 1/2 to 3/4 in the retirement status variable. ^c^Retirement defined by transition from 1/2/3 to 4 in the retirement status variable. ^e^Primary/secondary = 0, Tertiary = 1. ^f^No partner = 0, With partner = 1. ^g^Employed = 0, Unemployed = 1.

**Table S5**

*Sensitivity analysis with varying definitions of retirement (pension withdrawal, subjective retirement, stop working) in the effects on quality of life*

|  | Quality of life | | | | | | | | |
| --- | --- | --- | --- | --- | --- | --- | --- | --- | --- |
|  | Pension withdrawal^a^ | | | Subjective retirement^b^ | | | Stop working^c^ | | |
|  | Est. | 95% CI | *p* | Est. | 95% CI | *p* | Est. | 95% CI | *p* |
| *Fixed effects* | | | | | | | | | |
| Intercept | -0.09 | -0.14, -0.04 | <.001 | -0.08 | -0.13, -0.03 | .001 | -0.09 | -0.13, -0.04 | <.001 |
| Age at baseline | -0.02 | -0.03, -0.003 | .022 | -0.01 | -0.03, 0.002 | .104 | -0.01 | -0.02, 0.003 | .148 |
| Age (time-varying) | 0.01 | 0.01, 0.02 | <.001 | 0.01 | 0.01, 0.02 | <.001 | 0.01 | 0.01, 0.02 | <.001 |
| Retirement WP | 0.11 | 0.09, 0.14 | <.001 | 0.12 | 0.10, 0.14 | <.001 | 0.13 | 0.10, 0.15 | <.001 |
| Retirement BP | 0.13 | 0.03, 0.22 | .011 | 0.04 | -0.03, 0.12 | .273 | 0.02 | -0.05, 0.09 | .593 |
| Gender^d^ | 0.05 | 0.005, 0.10 | .030 | 0.05 | 0.01, 0.10 | .028 | 0.05 | 0.01, 0.10 | .021 |
| Education^e^ | 0.07 | 0.03, 0.12 | .003 | 0.07 | 0.02, 0.12 | .004 | 0.06 | 0.02, 0.11 | .008 |
| Relationship status^f^ | 0.21 | 0.17, 0.24 | <.001 | 0.21 | 0.18, 0.24 | <.001 | 0.21 | 0.18, 0.24 | <.001 |
| Employment status at baseline^g^ | -0.67 | -0.76, -0.59 | <.001 | -0.69 | -0.77, -0.60 | <.001 | -0.69 | -0.77, -0.60 | <.001 |
| Retirement WP × Age | -0.02 | -0.03, -0.01 | <.001 | -0.04 | -0.05, -0.03 | <.001 | -0.04 | -0.05, -0.03 | <.001 |
| Retirement BP × Age | -0.06 | -0.07, -0.05 | <.001 | -0.06 | -0.06, -0.05 | <.001 | -0.05 | -0.05, -0.04 | <.001 |
| *Random effects (SD)* | | | | | | | | | |
| Intercept | 0.82 | | | 0.82 | | | 0.82 | | |
| Age | 0.07 | | | 0.06 | | | 0.06 | | |
| Retirement WP | 0.32 | | | 0.31 | | | 0.30 | | |
| Residual | 0.47 | | | 0.47 | | | 0.47 | | |
| AIC | 61,622.50 | | | 61,539.72 | | | 61,674.18 | | |
| BIC | 61,773.37 | | | 61,690.59 | | | 61,825.05 | | |

*Note*. Dependent variable standardized (z-score) on baseline distribution to facilitate interpretation. WP = within-person effect, BP = between-person effect, AIC = Akaike Information Criterion, BIC = Bayesian Information Criterion, CI = confidence interval. ^a^Retirement defined by transition from 1 to 2/3/4 in the retirement status variable. ^b^Retirement defined by transition from 1/2 to 3/4 in the retirement status variable. ^c^Retirement defined by transition from 1/2/3 to 4 in the retirement status variable. ^e^Primary/secondary = 0, Tertiary = 1. ^f^No partner = 0, With partner = 1. ^g^Employed = 0, Unemployed = 1.

**Table S6**

*Sensitivity analysis with varying definitions of retirement (pension withdrawal, subjective retirement, stop working) in the effects on depressive symptoms*

|  | Depressive symptoms | | | | | | | | |
| --- | --- | --- | --- | --- | --- | --- | --- | --- | --- |
|  | Pension withdrawal^a^ | | | Subjective retirement^b^ | | | Stop working^c^ | | |
|  | Est. | 95% CI | *p* | Est. | 95% CI | *p* | Est. | 95% CI | *p* |
| *Fixed effects* | | | | | | | | | |
| Intercept | 0.15 | 0.11, 0.20 | <.001 | 0.15 | 0.10, 0.20 | <.001 | 0.16 | 0.11, 0.20 | <.001 |
| Age at baseline | -0.01 | -0.02, 0.01 | .334 | -0.01 | -0.02, 0.01 | .269 | -0.01 | -0.02, 0.00 | .125 |
| Age (time-varying) | 0.004 | <-0.001, 0.01 | .068 | 0.003 | -0.001, 0.01 | .109 | 0.003 | -0.001, 0.01 | .138 |
| Retirement WP | -0.10 | -0.13,  -0.07 | <.001 | -0.11 | -0.13,  -0.08 | <.001 | -0.12 | -0.14,  -0.09 | <.001 |
| Retirement BP | -0.08 | -0.17, 0.01 | .084 | -0.06 | -0.13, 0.01 | .101 | -0.03 | -0.10, 0.03 | .310 |
| Gender^d^ | 0.07 | 0.02, 0.11 | .002 | 0.06 | 0.02, 0.11 | .003 | 0.06 | 0.02, 0.10 | .005 |
| Education^e^ | -0.02 | -0.07, 0.02 | .287 | -0.02 | -0.07, 0.02 | .303 | -0.02 | -0.06, 0.02 | .395 |
| Relationship status^f^ | -0.37 | -0.41,  -0.34 | <.001 | -0.38 | -0.41,  -0.34 | <.001 | -0.37 | -0.41,  -0.34 | <.001 |
| Employment status at baseline^g^ | 0.62 | 0.53, 0.70 | <.001 | 0.62 | 0.54, 0.70 | <.001 | 0.62 | 0.54, 0.70 | <.001 |
| Retirement WP × Age | 0.02 | 0.01, 0.03 | <.001 | 0.04 | 0.03, 0.05 | <.001 | 0.04 | 0.04, 0.05 | <.001 |
| Retirement BP × Age | 0.05 | 0.04, 0.06 | <.001 | 0.04 | 0.03, 0.05 | <.001 | 0.03 | 0.03, 0.04 | <.001 |
| *Random effects (SD)* | | | | | | | | | |
| Intercept | 0.74 | | | 0.74 | | | 0.74 | | |
| Age | 0.07 | | | 0.07 | | | 0.07 | | |
| Retirement WP | 0.33 | | | 0.26 | | | 0.28 | | |
| Residual | 0.54 | | | 0.54 | | | 0.54 | | |
| AIC | 68,091.51 | | | 68,080.38 | | | 68,152.12 | | |
| BIC | 68,242.56 | | | 68,231.43 | | | 68,303.17 | | |

*Note*. Dependent variable standardized (z-score) on baseline distribution to facilitate interpretation. WP = within-person effect, BP = between-person effect, AIC = Akaike Information Criterion, BIC = Bayesian Information Criterion, CI = confidence interval. ^a^Retirement defined by transition from 1 to 2/3/4 in the retirement status variable. ^b^Retirement defined by transition from 1/2 to 3/4 in the retirement status variable. ^c^Retirement defined by transition from 1/2/3 to 4 in the retirement status variable. ^e^Primary/secondary = 0, Tertiary = 1. ^f^No partner = 0, With partner = 1. ^g^Employed = 0, Unemployed = 1.

**Table S7**

*Sensitivity analysis with varying definitions of retirement (pension withdrawal, subjective retirement, stop working) in the effects on disease burden*

|  | Disease burden | | | | | | | | |
| --- | --- | --- | --- | --- | --- | --- | --- | --- | --- |
|  | Pension withdrawal^a^ | | | Subjective retirement^b^ | | | Stop working^c^ | | |
|  | Est. | 95% CI | *p* | Est. | 95% CI | *p* | Est. | 95% CI | *p* |
| *Fixed effects* | | | | | | | | | |
| Intercept | 0.11 | 0.06, 0.16 | <.001 | 0.11 | 0.06, 0.16 | <.001 | 0.12 | 0.06, 0.17 | <.001 |
| Age at baseline | -0.04 | -0.05,  -0.02 | <.001 | -0.04 | -0.05,  -0.02 | <.001 | -0.04 | -0.05, -0.02 | <.001 |
| Age (time-varying) | 0.04 | 0.04, 0.04 | <.001 | 0.04 | 0.04, 0.04 | <.001 | 0.04 | 0.04, 0.04 | <.001 |
| Retirement WP | -0.05 | -0.07,  -0.02 | <.001 | -0.05 | -0.08,  -0.03 | <.001 | -0.06 | -0.09, -0.04 | <.001 |
| Retirement BP | 0.06 | -0.05, 0.17 | .272 | 0.09 | -0.001, 0.18 | .051 | 0.10 | 0.02, 0.17 | .015 |
| Gender^d^ | 0.03 | -0.02, 0.08 | .266 | 0.02 | -0.03, 0.07 | .369 | 0.02 | -0.03, 0.07 | .470 |
| Education^e^ | -0.27 | -0.32,  -0.22 | <.001 | -0.27 | -0.32,  -0.22 | <.001 | -0.26 | -0.31, -0.21 | <.001 |
| Relationship status^f^ | -0.05 | -0.08,  -0.01 | .007 | -0.05 | -0.08,  -0.01 | .008 | -0.05 | -0.08, -0.01 | .007 |
| Employment status at baseline^g^ | 0.81 | 0.71, 0.91 | <.001 | 0.81 | 0.71, 0.90 | <.001 | 0.80 | 0.70, 0.90 | <.001 |
| Retirement WP × Age | 0.01 | -0.002, 0.02 | .107 | 0.02 | 0.01, 0.02 | <.001 | 0.02 | 0.01, 0.03 | <.001 |
| Retirement BP × Age | 0.02 | 0.01, 0.03 | .002 | 0.02 | 0.01, 0.03 | <.001 | 0.01 | 0.004, 0.02 | .004 |
| *Random effects (SD)* | | | | | | | | | |
| Intercept | 0.87 | | | 0.87 | | | 0.87 | | |
| Age | 0.07 | | | 0.07 | | | 0.07 | | |
| Retirement WP | 0.25 | | | 0.24 | | | 0.25 | | |
| Residual | 0.40 | | | 0.40 | | | 0.40 | | |
| AIC | 43,463.03 | | | 43,451.67 | | | 43,521.63 | | |
| BIC | 43,609.22 | | | 43,597.86 | | | 43,667.82 | | |

*Note*. Dependent variable standardized (z-score) on baseline distribution to facilitate interpretation. WP = within-person effect, BP = between-person effect, AIC = Akaike Information Criterion, BIC = Bayesian Information Criterion, CI = confidence interval. ^a^Retirement defined by transition from 1 to 2/3/4 in the retirement status variable. ^b^Retirement defined by transition from 1/2 to 3/4 in the retirement status variable. ^c^Retirement defined by transition from 1/2/3 to 4 in the retirement status variable. ^e^Primary/secondary = 0, Tertiary = 1. ^f^No partner = 0, With partner = 1. ^g^Employed = 0, Unemployed = 1.

**Table S8**

*Sensitivity analysis with varying definitions of retirement (pension withdrawal, subjective retirement, stop working) in the effects on reasoning ability*

|  | Reasoning ability | | | | | | | | |
| --- | --- | --- | --- | --- | --- | --- | --- | --- | --- |
|  | Pension withdrawal^a^ | | | Subjective retirement^b^ | | | Stop working^c^ | | |
|  | Est. | 95% CI | *p* | Est. | 95% CI | *p* | Est. | 95% CI | *p* |
| *Fixed effects* | | | | | | | | | |
| Intercept | <0.001 | -0.06, 0.06 | .999 | -0.005 | -0.06, 0.05 | .869 | -0.01 | -0.06, 0.05 | .818 |
| Age at baseline | -0.13 | -0.15,  -0.12 | <.001 | -0.14 | -0.15,  -0.12 | <.001 | -0.13 | -0.14, -0.11 | <.001 |
| Age (time-varying) | 0.06 | 0.06, 0.07 | <.001 | 0.06 | 0.05, 0.07 | <.001 | 0.06 | 0.05, 0.06 | <.001 |
| Retirement WP | 0.01 | -0.03, 0.05 | .473 | 0.04 | 0.004, 0.08 | .030 | 0.04 | 0.01, 0.08 | .025 |
| Retirement BP | -0.01 | -0.12, 0.11 | .886 | 0.01 | -0.08, 0.10 | .766 | -0.07 | -0.15, 0.01 | .073 |
| Gender^d^ | -0.22 | -0.27, -0.18 | <.001 | -0.22 | -0.27,  -0.18 | <.001 | -0.22 | -0.26, -0.17 | <.001 |
| Education^e^ | 0.30 | 0.25, 0.34 | <.001 | 0.30 | 0.25, 0.35 | <.001 | 0.29 | 0.24, 0.34 | <.001 |
| Relationship status^f^ | 0.05 | <-0.001, 0.09 | .050 | 0.05 | <-0.001, 0.09 | .051 | 0.05 | 0.003, 0.10 | .038 |
| Employment status at baseline^g^ | -0.14 | -0.24,  -0.04 | .005 | -0.14 | -0.24, -0.04 | .005 | -0.13 | -0.23, -0.04 | .007 |
| Retirement WP × Age | -0.03 | -0.05,  -0.02 | <.001 | -0.03 | -0.04, -0.02 | <.001 | -0.02 | -0.03, -0.01 | .001 |
| Retirement BP × Age | -0.01 | -0.03, 0.01 | .376 | 0.004 | -0.01, 0.02 | .615 | 0.002 | -0.01, 0.01 | .768 |
| *Random effects (SD)* | | | | | | | | | |
| Intercept | 0.68 | | | 0.68 | | | 0.68 | | |
| Age | 0.04 | | | 0.05 | | | 0.05 | | |
| Retirement WP | 0.10 | | | 0.03 | | | 0.11 | | |
| Residual | 0.69 | | | 0.69 | | | 0.69 | | |
| AIC | 52,891.78 | | | 52,891.08 | | | 52,967.53 | | |
| BIC | 53,035.50 | | | 53,034.80 | | | 53,111.24 | | |

*Note*. Dependent variable standardized (z-score) on baseline distribution to facilitate interpretation. WP = within-person effect, BP = between-person effect, AIC = Akaike Information Criterion, BIC = Bayesian Information Criterion, CI = confidence interval. ^a^Retirement defined by transition from 1 to 2/3/4 in the retirement status variable. ^b^Retirement defined by transition from 1/2 to 3/4 in the retirement status variable. ^c^Retirement defined by transition from 1/2/3 to 4 in the retirement status variable. ^e^Primary/secondary = 0, Tertiary = 1. ^f^No partner = 0, With partner = 1. ^g^Employed = 0, Unemployed = 1.

**Table S9**

*Sensitivity analysis with varying definitions of retirement (pension withdrawal, subjective retirement, stop working) in the effects on memory*

|  | Memory | | | | | | | | |
| --- | --- | --- | --- | --- | --- | --- | --- | --- | --- |
|  | Pension withdrawal^a^ | | | Subjective retirement^b^ | | | Stop working^c^ | | |
|  | Est. | 95% CI | *p* | Est. | 95% CI | *p* | Est. | 95% CI | *p* |
| *Fixed effects* | | | | | | | | | |
| Intercept | 0.01 | -0.05, 0.07 | .779 | 0.01 | -0.05, 0.06 | .842 | 0.01 | -0.05, 0.07 | .826 |
| Age at baseline | -0.09 | -0.11, -0.08 | <.001 | -0.09 | -0.11, -0.08 | <.001 | -0.09 | -0.10, -0.08 | <.001 |
| Age (time-varying) | 0.07 | 0.07, 0.08 | <.001 | 0.07 | 0.06, 0.07 | <.001 | 0.07 | 0.07, 0.08 | <.001 |
| Retirement WP | 0.01 | -0.03, 0.05 | .508 | 0.08 | 0.05, 0.12 | <.001 | 0.08 | 0.04, 0.12 | <.001 |
| Retirement BP | 0.03 | -0.08, 0.14 | .614 | 0.10 | 0.01, 0.18 | .025 | 0.06 | -0.01, 0.13 | .116 |
| Gender^d^ | 0.19 | 0.15, 0.24 | <.001 | 0.19 | 0.14, 0.23 | <.001 | 0.19 | 0.15, 0.24 | <.001 |
| Education^e^ | 0.24 | 0.19, 0.29 | <.001 | 0.25 | 0.20, 0.29 | <.001 | 0.24 | 0.19, 0.29 | <.001 |
| Relationship status^f^ | -0.004 | -0.05, 0.05 | .878 | -0.004 | -0.05, 0.04 | .873 | -0.004 | -0.05, 0.05 | .886 |
| Employment status at baseline^g^ | -0.18 | -0.28, -0.08 | <.001 | -0.19 | -0.28, -0.09 | <.001 | -0.18 | -0.28, -0.09 | <.001 |
| Retirement WP × Age | -0.05 | -0.07, -0.04 | <.001 | -0.04 | -0.06, -0.03 | <.001 | -0.05 | -0.06, -0.04 | <.001 |
| Retirement BP × Age | -0.01 | -0.03, 0.01 | .180 | -0.01 | -0.02, 0.01 | .223 | -0.02 | -0.03, -0.003 | .019 |
| *Random effects (SD)* | | | | | | | | | |
| Intercept | 0.70 | | | 0.69 | | | 0.70 | | |
| Age | 0.07 | | | 0.07 | | | 0.06 | | |
| Retirement WP | 0.06 | | | 0.15 | | | 0.03 | | |
| Residual | 0.66 | | | 0.66 | | | 0.66 | | |
| AIC | 43,351.58 | | | 43,340.64 | | | 43,208.39 | | |
| BIC | 43,491.50 | | | 43,480.56 | | | 43,548.31 | | |

*Note*. Dependent variable standardized (z-score) on baseline distribution to facilitate interpretation. WP = within-person effect, BP = between-person effect, AIC = Akaike Information Criterion, BIC = Bayesian Information Criterion, CI = confidence interval. ^a^Retirement defined by transition from 1 to 2/3/4 in the retirement status variable. ^b^Retirement defined by transition from 1/2 to 3/4 in the retirement status variable. ^c^Retirement defined by transition from 1/2/3 to 4 in the retirement status variable. ^e^Primary/secondary = 0, Tertiary = 1. ^f^No partner = 0, With partner = 1. ^g^Employed = 0, Unemployed = 1.

**Table S10**

*Interaction effects of retirement and age by job satisfaction on life satisfaction, quality of life, depressive symptoms, disease burden, reasoning ability, and memory*

|  | Life satisfaction | | | Quality of life | | | Depressive symptoms | | | Disease burden | | | Reasoning ability | | | Memory | | |
| --- | --- | --- | --- | --- | --- | --- | --- | --- | --- | --- | --- | --- | --- | --- | --- | --- | --- | --- |
|  | Est. | 95% CI | *p* | Est. | 95% CI | *p* | Est. | 95% CI | *p* | Est. | 95% CI | *p* | Est. | 95% CI | *p* | Est. | 95% CI | *p* |
| *Fixed effects* | | | | | | | | | | | | | | | | | | |
| Intercept | -0.18 | -0.23,  -0.12 | <.001 | -0.01 | -0.07, 0.04 | .613 | 0.07 | 0.02, 0.12 | .007 | 0.09 | 0.03, 0.15 | .002 | 0.01 | -0.06, 0.08 | .719 | 0.05 | -0.02, 0.12 | .173 |
| Age at baseline | -0.06 | -0.07,  -0.04 | <.001 | -0.05 | -0.06,  -0.03 | <.001 | 0.02 | 0.01, 0.03 | .004 | -0.04 | -0.05,  -0.02 | <.001 | -0.13 | -0.14,  -0.11 | <.001 | -0.10 | -0.12,  -0.08 | <.001 |
| Age (time-varying) | 0.02 | 0.02, 0.03 | <.001 | 0.01 | 0.01, 0.01 | <.001 | 0.005 | <0.001, 0.01 | .044 | 0.04 | 0.04, 0.05 | <.001 | 0.06 | 0.05, 0.07 | <.001 | 0.07 | 0.06, 0.07 | <.001 |
| Retirement WP | 0.04 | 0.03, 0.04 | <.001 | 0.04 | 0.03, 0.05 | <.001 | -0.04 | -0.05,  -0.03 | <.001 | -0.02 | -0.03,  -0.01 | <.001 | 0.01 | -0.003, 0.03 | .112 | 0.03 | 0.01, 0.05 | <.001 |
| Retirement BP | 0.12 | 0.09, 0.15 | <.001 | 0.09 | 0.05, 0.12 | <.001 | -0.08 | -0.11,  -0.05 | <.001 | 0.01 | -0.03, 0.05 | .707 | -0.004 | -0.05, 0.04 | .849 | 0.05 | 0.02, 0.09 | .006 |
| Gender^a^ | 0.02 | -0.03, 0.07 | .447 | 0.03 | -0.02, 0.07 | .304 | 0.08 | 0.03, 0.12 | .001 | -0.01 | -0.06, 0.05 | .754 | -0.23 | -0.29,  -0.18 | <.001 | 0.16 | 0.11, 0.22 | <.001 |
| Education^b^ | 0.05 | -0.003, 0.09 | .069 | 0.04 | -0.01, 0.09 | .111 | 0.01 | -0.03, 0.06 | .541 | -0.24 | -0.30,  -0.19 | <.001 | 0.32 | 0.26, 0.37 | <.001 | 0.28 | 0.23, 0.34 | <.001 |
| Relationship status^c^ | 0.31 | 0.27, 0.35 | <.001 | 0.16 | 0.13, 0.20 | <.001 | -0.31 | -0.35,  -0.27 | <.001 | -0.04 | -0.08, 0.005 | .086 | 0.04 | -0.02, 0.09 | .154 | -0.04 | -0.09, 0.02 | .218 |
| Employment status at baseline^d^ | -0.44 | -0.60,  -0.28 | <.001 | -0.49 | -0.65,  -0.33 | <.001 | 0.46 | 0.31, 0.60 | <.001 | 0.50 | 0.32, 0.68 | <.001 | 0.06 | -0.12, 0.24 | .513 | -0.03 | -0.21, 0.15 | .777 |
| Retirement WP × Age | -0.004 | -0.01,  -0.001 | .012 | -0.01 | -0.01,  -0.01 | <.001 | 0.01 | 0.01, 0.01 | <.001 | 0.004 | 0.001, 0.01 | .007 | -0.01 | -0.02,  -0.01 | <.001 | -0.02 | -0.03,  -0.02 | <.001 |
| Retirement BP × Age | -0.01 | -0.01,  -0.01 | <.001 | -0.02 | -0.02,  -0.01 | <.001 | 0.01 | 0.01, 0.02 | <.001 | 0.01 | 0.001, 0.01 | .014 | -0.003 | -0.01, 0.004 | .339 | -0.01 | -0.02,  -0.003 | .005 |
| Job satisfaction | 0.30 | 0.28, 0.33 | <.001 | 0.33 | 0.31, 0.36 | <.001 | -0.25 | -0.28,  -0.23 | <.001 | -0.15 | -0.18,  -0.12 | <.001 | -0.001 | -0.03, 0.03 | .933 | 0.01 | -0.02, 0.04 | .437 |
| Job satisfaction × Age | 0.002 | -0.002, 0.01 | .242 | -0.002 | -0.01, 0.002 | .354 | 0.001 | -0.003, 0.01 | .577 | 0.001 | -0.003, 0.01 | .529 | -0.001 | -0.01, 0.01 | .864 | -0.001 | -0.01, 0.01 | .865 |
| Job satisfaction × Retirement WP | -0.03 | -0.04,  -0.02 | <.001 | -0.03 | -0.04,  -0.02 | <.001 | 0.03 | 0.02, 0.04 | <.001 | 0.01 | -0.002, 0.02 | .152 | 0.002 | -0.01, 0.02 | .775 | 0.01 | -0.01, 0.02 | .379 |
| Job satisfaction × Age × Retirement WP | 0.001 | -0.002, 0.004 | .669 | -0.002 | -0.005, 0.002 | .323 | <-0.001 | -0.004, 0.003 | .870 | <-0.001 | -0.004, 0.003 | .928 | -0.001 | -0.01, 0.01 | .797 | -0.002 | -0.01, 0.003 | .497 |
| *Random effects (SD)* | | | | | | | | | | | | | | | | | | |
| Intercept | 0.71 | | | 0.72 | | | 0.65 | | | 0.81 | | | 0.67 | | | 0.68 | | |
| Age | 0.06 | | | 0.06 | | | 0.06 | | | 0.06 | | | 0.06 | | | 0.06 | | |
| Retirement WP | 0.12 | | | 0.12 | | | 0.11 | | | 0.10 | | | 0.08 | | | 0.03 | | |
| Residual | 0.47 | | | 0.47 | | | 0.53 | | | 0.39 | | | 0.68 | | | 0.66 | | |
| AIC | 45,127.95 | | | 43,375.15 | | | 47,700.94 | | | 31,279.58 | | | 39,661.64 | | | 31,767.47 | | |
| BIC | 45,305.99 | | | 43,552.57 | | | 47,878.61 | | | 31,451.73 | | | 39,831.06 | | | 31,931.89 | | |
| *N* / observations | 3,831 / 24,171 | | | 3,816 / 23,493 | | | 3,865 / 23,762 | | | 3,675 / 18,489 | | | 3,193 / 16,332 | | | 3,339 / 13,010 | | |

*Note*. Dependent variables standardized (z-score) on baseline distribution to facilitate interpretation. WP = within-person effect, BP = between-person effect, AIC = Akaike Information Criterion, BIC = Bayesian Information Criterion, CI = confidence interval. ^a^Male = 0, Female = 1. ^b^Primary/secondary = 0, Tertiary = 1. ^c^No partner = 0, With partner = 1. ^d^Employed = 0, Unemployed = 1.

**Table S11**

*Interaction effects of retirement and age by involuntary transition on life satisfaction, quality of life, depressive symptoms, disease burden, reasoning ability, and memory*

|  | Life satisfaction | | | Quality of life | | | Depressive symptoms | | | Disease burden | | | Reasoning ability | | | Memory | | |
| --- | --- | --- | --- | --- | --- | --- | --- | --- | --- | --- | --- | --- | --- | --- | --- | --- | --- | --- |
|  | Est. | 95% CI | *p* | Est. | 95% CI | *p* | Est. | 95% CI | *p* | Est. | 95% CI | *p* | Est. | 95% CI | *p* | Est. | 95% CI | *p* |
| *Fixed effects* | | | | | | | | | | | | | | | | | | |
| Intercept | -0.06 | -0.12,  -0.01 | .026 | 0.07 | 0.01, 0.12 | .017 | 0.002 | -0.05, 0.06 | .942 | -0.01 | -0.07, 0.05 | .731 | 0.02 | -0.04, 0.09 | .509 | 0.04 | -0.03, 0.11 | .251 |
| Age at baseline | -0.02 | -0.03,  -0.002 | .025 | -0.01 | -0.02, 0.01 | .352 | -0.01 | -0.03, 0.002 | .091 | -0.05 | -0.07,  -0.03 | <.001 | -0.12 | -0.14,  -0.10 | <.001 | -0.09 | -0.10,  -0.07 | <.001 |
| Age (time-varying) | 0.02 | 0.01, 0.02 | <.001 | 0.01 | 0.005, 0.01 | <.001 | 0.01 | 0.004, 0.01 | .001 | 0.04 | 0.04, 0.05 | <.001 | 0.06 | 0.05, 0.06 | <.001 | 0.07 | 0.06, 0.08 | <.001 |
| Retirement WP | 0.05 | 0.04, 0.06 | <.001 | 0.05 | 0.04, 0.06 | <.001 | -0.05 | -0.07,  -0.04 | <.001 | -0.03 | -0.04,  -0.02 | <.001 | 0.02 | 0.01, 0.04 | .007 | 0.03 | 0.01, 0.04 | .001 |
| Retirement BP | 0.08 | 0.04, 0.12 | <.001 | 0.03 | -0.01, 0.07 | .214 | -0.01 | -0.05, 0.02 | .442 | 0.06 | 0.02, 0.11 | .005 | -0.04 | -0.08, 0.004 | .075 | -0.003 | -0.05, 0.04 | .897 |
| Gender^a^ | 0.04 | -0.01, 0.09 | .082 | 0.07 | 0.02, 0.12 | .005 | 0.05 | 0.01, 0.10 | .019 | 0.01 | -0.04, 0.07 | .673 | -0.23 | -0.27,  -0.18 | <.001 | 0.19 | 0.14, 0.24 | <.001 |
| Education^b^ | 0.07 | 0.02, 0.11 | .008 | 0.05 | -0.001, 0.10 | .056 | -0.02 | -0.06, 0.03 | .465 | -0.25 | -0.30,  -0.19 | <.001 | 0.30 | 0.24, 0.35 | <.001 | 0.23 | 0.18, 0.28 | <.001 |
| Relationship status^c^ | 0.31 | 0.27, 0.34 | <.001 | 0.18 | 0.15, 0.22 | <.001 | -0.34 | -0.38,  -0.30 | <.001 | -0.04 | -0.07, 0.002 | .064 | 0.05 | 0.002, 0.10 | .040 | -0.002 | -0.06, 0.05 | .950 |
| Employment status at baseline^d^ | -0.31 | -0.40,  -0.21 | <.001 | -0.35 | -0.45,  -0.25 | <.001 | 0.31 | 0.22, 0.40 | <.001 | 0.58 | 0.47, 0.69 | <.001 | -0.09 | -0.19, 0.02 | .123 | -0.19 | -0.30,  -0.08 | .001 |
| Retirement WP × Age | -0.01 | -0.01,  -0.002 | .005 | -0.01 | -0.02,  -0.01 | <.001 | 0.01 | 0.01, 0.02 | <.001 | 0.01 | 0.002, 0.01 | .005 | -0.01 | -0.02,  -0.004 | .002 | -0.02 | -0.02,  -0.01 | <.001 |
| Retirement BP × Age | -0.01 | -0.02,  -0.01 | <.001 | -0.02 | -0.02,  -0.02 | <.001 | 0.01 | 0.01, 0.02 | <.001 | 0.003 | -0.001, 0.01 | .145 | 0.005 | -0.002, 0.01 | .160 | -0.001 | -0.01, 0.01 | .870 |
| Involuntary transition | -0.44 | -0.48,  -0.40 | <.001 | -0.46 | -0.51,  -0.42 | <.001 | 0.43 | 0.39, 0.47 | <.001 | 0.36 | 0.31, 0.41 | <.001 | -0.06 | -0.11,  -0.01 | .017 | -0.02 | -0.07, 0.03 | .377 |
| Involuntary transition × Age | 0.01 | 0.002, 0.02 | .004 | 0.005 | -0.002, 0.01 | .143 | -0.01 | -0.02,  -0.01 | <.001 | -0.003 | -0.01, 0.004 | .363 | -0.001 | -0.01, 0.01 | .822 | -0.01 | -0.02,  -0.002 | .023 |
| Involuntary transition × Retirement WP | -0.04 | -0.06,  -0.03 | <.001 | -0.03 | -0.04,  -0.01 | <.001 | 0.04 | 0.02, 0.05 | <.001 | 0.03 | 0.01, 0.04 | .001 | -0.01 | -0.04, 0.01 | .396 | -0.005 | -0.03, 0.02 | .706 |
| Involuntary transition × Age × Retirement WP | 0.003 | -0.002, 0.01 | .244 | -0.003 | -0.01, 0.003 | .329 | 0.004 | -0.002, 0.01 | .147 | -0.001 | -0.01, 0.005 | .748 | 0.001 | -0.01, 0.01 | .916 | -0.01 | -0.01, 0.003 | .205 |
| *Random effects (SD)* | | | | | | | | | | | | | | | | | | |
| Intercept | 0.76 | | | 0.78 | | | 0.71 | | | 0.85 | | | 0.69 | | | 0.71 | | |
| Age | 0.07 | | | 0.06 | | | 0.07 | | | 0.07 | | | 0.05 | | | 0.07 | | |
| Retirement WP | 0.12 | | | 0.12 | | | 0.12 | | | 0.10 | | | 0.05 | | | 0.01 | | |
| Residual | 0.48 | | | 0.47 | | | 0.54 | | | 0.40 | | | 0.69 | | | 0.66 | | |
| AIC | 56,053.90 | | | 53,446.23 | | | 59,329.65 | | | 38,021.82 | | | 47,308.77 | | | 38,940.70 | | |
| BIC | 56,236.24 | | | 53,627.94 | | | 59,511.49 | | | 38,197.79 | | | 47,481.97 | | | 38,108.89 | | |
| *N* / observations | 4,459 / 29,389 | | | 4,454 / 28,544 | | | 4,466 / 28,718 | | | 4,305 / 21,966 | | | 3,754 / 19,396 | | | 3,839 / 15,441 | | |

*Note*. Dependent variables standardized (z-score) on baseline distribution to facilitate interpretation. WP = within-person effect, BP = between-person effect, AIC = Akaike Information Criterion, BIC = Bayesian Information Criterion, CI = confidence interval. ^a^Male = 0, Female = 1. ^b^Primary/secondary = 0, Tertiary = 1. ^c^No partner = 0, With partner = 1. ^d^Employed = 0, Unemployed = 1.
